# Brownian processes in human motor control support descending neural velocity commands

**DOI:** 10.1038/s41598-024-58380-5

**Published:** 2024-04-09

**Authors:** Federico Tessari, James Hermus, Rika Sugimoto-Dimitrova, Neville Hogan

**Affiliations:** 1 Department of Mechanical Engineering, Massachusetts Institute of Technology, Cambridge, MA USA; 2 Department of Brain and Cognitive Sciences, Massachusetts Institute of Technology, Cambridge, MA USA

**Keywords:** Neuroscience, Motor control

## Abstract

The motor neuroscience literature suggests that the central nervous system may encode some motor commands in terms of velocity. In this work, we tackle the question: what consequences would velocity commands produce at the behavioral level? Considering the ubiquitous presence of noise in the neuromusculoskeletal system, we predict that velocity commands affected by stationary noise would produce “random walks”, also known as Brownian processes, in position. Brownian motions are distinctively characterized by a linearly growing variance and a power spectral density that declines in inverse proportion to frequency. This work first shows that these Brownian processes are indeed observed in unbounded motion tasks e.g., rotating a crank. We further predict that such growing variance would still be present, but bounded, in tasks requiring a constant posture e.g., maintaining a static hand position or quietly standing. This hypothesis was also confirmed by experimental observations. A series of descriptive models are investigated to justify the observed behavior. Interestingly, one of the models capable of accounting for all the experimental results must feature forward-path velocity commands corrupted by stationary noise. The results of this work provide behavioral support for the hypothesis that humans plan the motion components of their actions in terms of velocity.

## Introduction

Quite aside from its obvious scientific importance, a quantitative understanding of how neural activity in the central nervous system (CNS) manages the coordination of perception and action would have considerable practical value. Beyond the diagnosis and treatment of neurological disorders lies the promise of brain-machine interfaces—new ways to restore human capability after impairment or to augment it before injury. Already some remarkable advances have been reported^1–3^. Notably, two successful demonstrations of controlling multi-joint robotic/prosthetic arms (with up to 22 degrees of freedom) interpreted the electrical signals recorded from the cerebral cortex as specifying velocity^4,5^. These studies reinforced compelling earlier demonstrations that complex drawing motions could be extracted from recordings of cortical neural activity that were interpreted as velocity commands in Schwartz et al.^6–9^.

Stochastic variation or ‘noise’ is ubiquitous in the neuromotor system (and in measurements of neural activity). The behavioral consequences of that noise depend sensitively on the neuromotor control architecture and how the noise is expressed in the neuro-mechanical system. The contribution of this paper is to report behavioral observations of the stochastic processes in human motor control and articulate model classes that can and cannot account for this behavior.

### Why command velocity?

What might be the advantages of velocity commands? Engineering may provide insight:

One example comes from amputation prosthetic applications. For practical reasons (especially their limited acceptable weight and volume) typical myoelectrically-controlled upper-limb prostheses are actuated by small electric motors with high-ratio speed reduction, which amplifies output torque in proportion, enabling lighter, more compact designs^10–12^. The result is a non-back-drivable prosthesis quite different from natural limb behavior—external forces have minimal influence on prosthesis motion. By mapping myoelectric activity to (commanded) motor speed, maintaining a fixed posture (e.g. to carry a coat) is achieved by relaxing the controlling muscles. Fixed posture becomes a ‘default’ mode requiring no active intervention (and no unnecessary drain on batteries).

Another example is found in robot control. A typical task involves the motion of the robot end-effector (i.e. ‘hand’) but that motion is generated by actuators located at the robot’s degrees of freedom (i.e. ‘joints’). Joint motion uniquely defines task motion. However, execution requires mapping the planned task-space motion back to the corresponding joint-space motion. This requires inverting the kinematic relation between joint motion and task motion but that inversion often has no ‘closed-form’ representation. A practical alternative is to map task-space velocity to joint-space velocity^13^. This linear algebra calculation uses the Jacobian matrix of the kinematic map. While the inverse Jacobian may not be unique, it is easy to add conditions that yield a well-defined solution^14^.

Yet another example is found in aeronautic and aerospace applications. Flying requires control in six dimensions (three rotations and three translations). Among these dimensions, rotations provide a non-trivial challenge; a means to stabilize rotational motions was the essence of the Wright Brothers’ 1906 U.S. patent^15^. Finite angular rotations do not commute; the order in which they are performed affects a body’s resulting orientation. Unlike finite translations, they cannot simply be added as vectors. Remarkably, angular velocities are quite different: they commute (the order in which they are performed does not matter) and they may be added (linearly superimposed) as vectors. In practice, the most preferred representation of rotation for aircraft and spacecraft is via angular velocity relative to a body-fixed reference frame (roll, pitch, and yaw).

Of course, there is no guarantee that these engineering considerations apply to biology. Nevertheless, biology is subject to the same mechanical physics. If commanding velocity is a practical way to ‘work around’ the constraints of mechanical physics, we may expect related strategies in biology.

### Consequences of ubiquitous ‘noise’

Another important feature of the neuromotor system is the pervasive presence of stochasticity or ‘noise’^16,17^. Stochasticity is ubiquitous in both neurons and muscles. Electroencephalographic (EEG) and electromyographic (EMG) data are notoriously noisy. While some of this noise is due to limitations of the measurement technology, the dominant component is due to intrinsically stochastic neuro-muscular behavior. The main sources of noise in the neuromotor system are three: (i) sensory noise, due to transduction and amplification of sensory signals; (ii) cellular noise, resulting from voltage-gated ion channels which leads to electrical noise and synaptic noise; and (iii) motor noise^16^. The variable that noise corrupts can have profound implications. In this work, we consider the behavioral consequences of velocity-level commands corrupted by stationary noise. If the central nervous system commands motion in terms of velocity, and those commands are affected by stationary noise, an immediate consequence is that noise on position would necessarily be non-stationary. For example, if the noise corrupting velocity commands is assumed to be white with constant strength (a common modeling assumption) the noise corrupting position would necessarily be Brownian^18^, a ‘random walk’ phenomenon characterized by variance (second central moment) that grows linearly with time^19^ and power spectral density (PSD) declining in inverse proportion to frequency (−20 dB/decade on a log–log Bode magnitude plot^18^).

To test this prediction would require experimental observation of unbounded variation; how might that be possible? Though it may seem counterintuitive, despite the bounded workspace of the human limbs—human joints all have a limited range of motion—it is possible to experience unbounded behavior in a finite workspace i.e., “the possibility of a finite and yet unbounded space”^20^. Turning a crank is a simple example: a crank-and-pedal arrangement is used in a typical bicycle; hand cranks are common in manually-operated machinery. While the foot (respectively hand) is confined to a finitely bounded range of distances from a stationary pelvis (respectively thorax), the angular displacement of the bicycle crank (respectively hand crank) is unbounded; in principle, infinite angular displacement is possible. The unbounded, thus infinite, rotation enabled by the crank provides a valuable experimental condition to test our hypothesis. In the following we report observations of apparently unbounded variance in the human behavior of turning a simple hand crank.

Conversely, there are situations in which unbounded behavior cannot be observed experimentally. Upright posture provides an example; to maintain stable quiet standing, the center of pressure (CoP) of foot-floor interaction forces must remain within the base of support defined by the feet. However, that physical bound does not require the noise to be stationary; CoP variance may grow or decline with time—i.e. ‘drift’ as in a random walk—provided it remains within the base of support. The literature on human postural behavior confirms the presence of drift in quiet standing: the CoP of foot-floor interaction meanders within the foot-defined base of support in a manner resembling a random walk^21,22^. Several descriptive models have been proposed to account for the ‘random walk’ nature of CoP variation^21–25^. If our posture roams following a random walk, how can we stand without falling? The presence of random walks, and thus a growing variance, would require individuals to trigger specific mechanisms to avoid failure.

In this work, we investigated three different tasks: crank turning, maintaining a static hand posture and maintaining upright posture. The crank-turning task provided an experimental design to verify the presence of unbounded growing variance. The two postural tasks provided instead a counterpart to assess the presence of the same random walks and investigate the underlying mechanisms adopted by humans to limit such processes. The decision to consider two postural tasks aimed to generalize our findings to whole-body motor control, rather than limiting our findings to either the upper or lower limbs.

Here we show that the non-stationary ‘random walk’ behavior can emerge in three distinct ways i.e., control architectures. We present new experimental evidence to support our predictions and show that only one of these three architectures is plausibly competent to account for all of the experimental evidence.

## Results

### Crank turning

**Figure 1:**
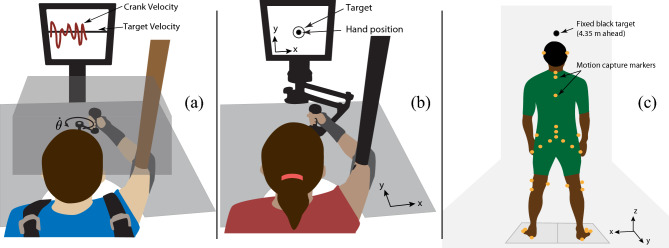
Experimental setups. (**a**) Crank turning: the crank was used to provide a circular constraint. Vision of the arm and crank was occluded but the subject was provided with visual speed feedback. The wrist was braced, the upper arm was supported by a sling, and the shoulders were strapped to a chair. (**b**) Hand posture: the InMotion2 robot was used to measure hand position. Visual feedback of the hand position was provided on a display. Vision of the arm and the robot was not occluded. The arm was supported by a sling. (**c**) Quiet standing^27^: subjects stood upright with their arms by their side, with their gaze fixed on a black target on a wall 4.35 m ahead. Motion capture markers were placed on their body. Further details of the three experiments are provided in the Material and Methods section.

A crank-turning experiment was conducted with 10 subjects (Fig. 1a). Crank turning was selected since it is particularly common in human activities of daily living^26^ but has the remarkable property of allowing unbounded motion despite the finite workspace of the human arm. Subjects were instructed to turn a crank at a constant speed in the clock-wise direction. Visual feedback of the rotational speed was provided. No explicit display of the angular position of the crank was provided, though normal proprioceptive feedback was available.

**Figure 2:**
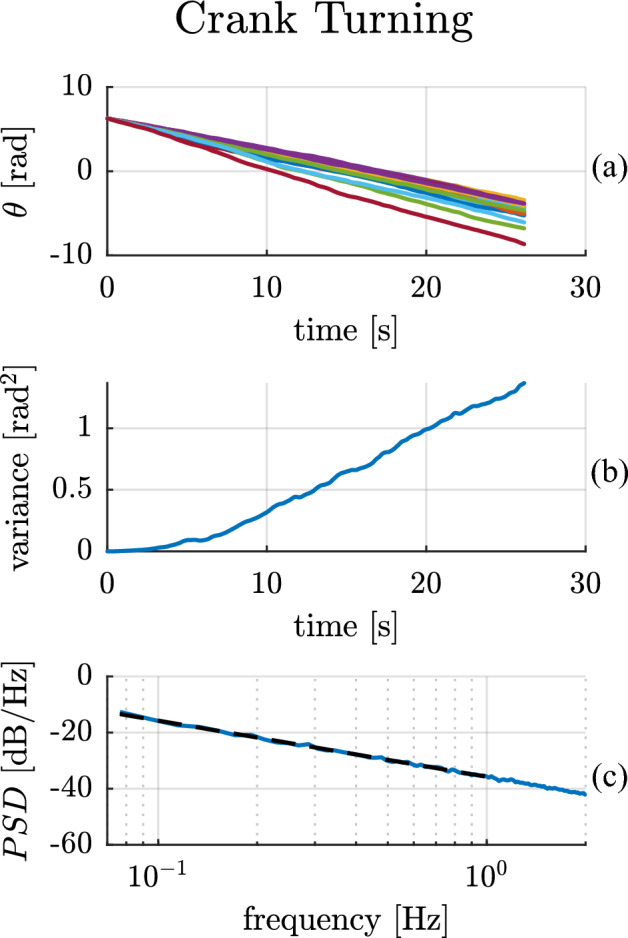
Crank turning experimental results for a single subject. The top panel (**a**) shows the crank angular position trajectory over time for multiple trials. The central panel (**b**) presents the position variance computed across trials. The bottom panel (**c**) shows a Bode magnitude plot of the average power spectral density of the angular position (solid blue line) compared to the best-fit line with a slope of −20.01 dB/dec (black dashed line).

The experimental results for a single subject performing the crank-turning task are presented in Fig. 2. The three panels show: (a) the time trajectories of the crank angle over multiple crank rotations, (b) the crank-angle variance, and (c) the Bode magnitude plot of the related power spectral density. The variance was computed across the ensemble (21 trials), while the PSD was computed for each trial and then averaged across trials.

The single-subject data in Fig. 2 exhibit Brownian behavior—an unbounded, linearly-growing variance of crank angle with time ($${R}_{var}^{2}=0.98$$). In the frequency domain, we expected a $$-20$$ dB/dec slope in the Bode magnitude plot of the crank angle, which we observed $$(-20.01 dB/dec$$, $${R}_{PSD}^{2}=0.997)$$, with 95% CI $$[-20.35 -19.67]$$ dB/dec at the lowest frequencies (0.07–1 Hz).

The same behavior was observed across all subjects, with an average ${R}_{var}^{2}=0.96\pm 0.02$ for the variance slope, and an average Bode magnitude slope of $-20.89$ dB/dec (${R}_{PSD}^{2}=0.993\pm 0.003)$ with 95% CI of $[-21.40 -20.36]$ dB/dec at low frequencies. The “Supplementary Information” contains ${R}_{var}^{2}$ and ${R}_{PSD}^{2}$ for the variance and PSD models and the 95% confidence intervals of the Bode magnitude slopes for all subjects.

### Hand posture

A postural task for the upper limb was devised (Fig. 1b) to test the presence of bounded Brownian-like behavior in a static positioning task. 10 subjects were asked to hold their hand at a fixed position for 4 min while visual feedback of hand position was provided. A sling supported the arm to minimize the gravitational load, while a robotic manipulandum with negligible friction was used to measure the planar (x,y) trajectory of the hand. 10 trials were performed.

**Figure 3:**
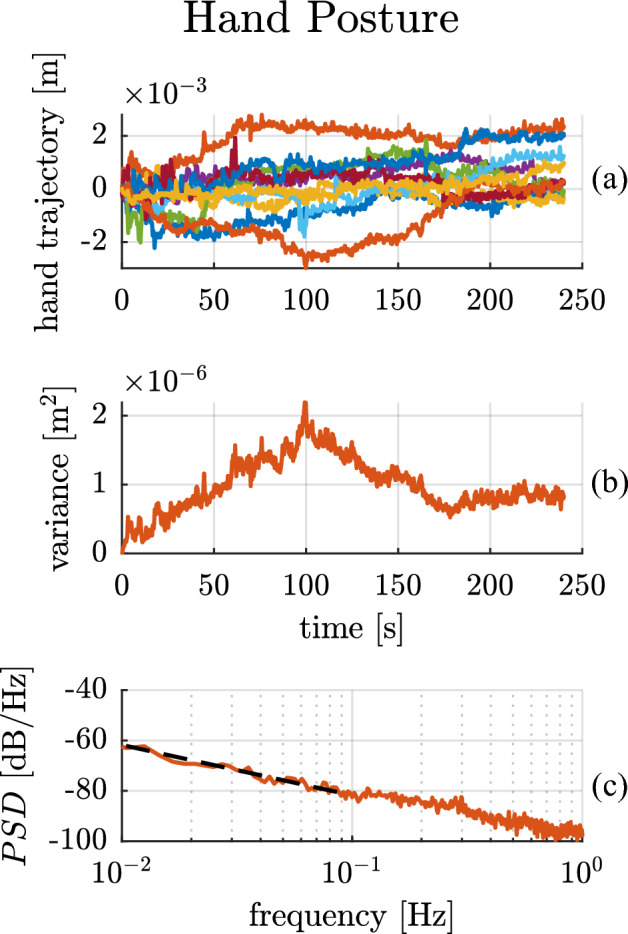
Hand posture experimental results of a single subject. The top panel (**a**) shows the trajectories of the ‘x’ Cartesian coordinate of the hand over time for multiple trials. The central panel (**b**) presents the position variance computed across trials for the ‘x’ direction. Note the obviously non-stationary character of the noise process. The bottom panel (**c**) shows a Bode magnitude plot of the average power spectral density of hand position in the ‘x’ direction (solid red line) compared to the best-fit line with a slope of −18.46 dB/dec (black dashed line).

The experimental results for a single subject performing the hand-posture task are presented in Fig. 3. The three panels show: (a) the time trajectories of hand position over the 10 trials, (b) the hand-position variance, and (c) the Bode magnitude plot of the PSD of hand position. The x-coordinate of the hand position is presented; the y-coordinate (omitted for clarity) exhibited similar behavior.

Once again, the single-subject data show that hand-position variance grew approximately linearly with time over the first minute in both the ‘x’ and ‘y’ directions $$({R}_{{x}_{var}}^{2}=0.93, {R}_{{y}_{var}}^{2}=0.95)$$, suggesting that the presence of a Brownian process is an intrinsic feature of human neuromotor behavior. However, unlike in the crank-turning task, the hand-position variance reached a ‘break point’ ($${t}_{b{p}_{x}}=108 s, {t}_{b{p}_{y}}=61 s)$$ after which the variance stopped its linear increase. In fact, linear regression of the variance computed over the whole time period showed a poor fit with $${R}_{{x}_{var}}^{2}=0, {R}_{{y}_{var}}^{2}=0.06$$. Over the low-frequency range (0.01–0.1 Hz) the Bode magnitude plot of the PSD displayed a $$-18.46$$ dB/dec slope ($${R}_{{x}_{PSD}}^{2}=0.98)$$, with 95% CI $$\left[-21.08 -16.84\right]$$ dB/dec in ‘x’ and a $$-21.39$$ dB/dec slope ($${R}_{{y}_{PSD}}^{2}=0.93)$$ with 95% CI $$\left[-23.16 -19.61\right]$$ dB/dec in ‘y’.

In all 10 subjects, a consistent linearly-growing variance up to a break point was observed, with an average ${R}_{var}^{2}=0.85\pm 0.1$. The break point between linear growth and bounded variance varied across subjects, ranging from a few seconds (1–5 s) to minutes (100–135 s). Subjects’ low-frequency PSD Bode magnitude plots presented good linear fits (${R}_{PSD}^{2}=0.86\pm 0.09)$, with an average best-fit slope of $-16.69$ dB/dec and an average 95% CI of $\left[-18.48 -14.90\right]$ dB/dec in both directions. The minimum frequency for the linear fit was 0.01 Hz for all subjects except subject no. 5 who had a minimum frequency for the linear fit of 0.04 Hz. The flattening of the low-frequency Bode magnitude slope may be attributed to the boundedness of the process, and was reproduced in simulation (see the Descriptive Model below). The “Supplementary Information” reports the variance and coefficients of determination ${R}_{var}^{2}$, ${R}_{PSD}^{2}$ and breakpoints, as well as the average PSD Bode magnitude slope and related 95% confidence intervals for all 10 subjects.

### Quiet standing

Upright standing posture has been a prevalent behavioral paradigm in the study of the stochastic processes underlying human motor control. To enable a comparison between the upper limb experiment presented above with behavior during natural upright posture, the same analysis was run on the experimental data collected by dos Santos et al.^27^. This database contains observations of 49 subjects standing for 60 s at a time (Fig. 1 right panel). Motion-capture markers were placed on the body to determine the center of mass (CoM) trajectory and joint angles over time.

In our study, the CoM trajectory in Cartesian coordinates during the normal quiet-standing trials (eyes open, standing on a flat rigid surface) for 26 young unimpaired subjects was considered. The CoM was chosen as a macroscopic representation of overall body behavior; it may be considered equivalent to the end-effector position of an open-kinematic-chain model of upright human posture.

**Figure 4:**
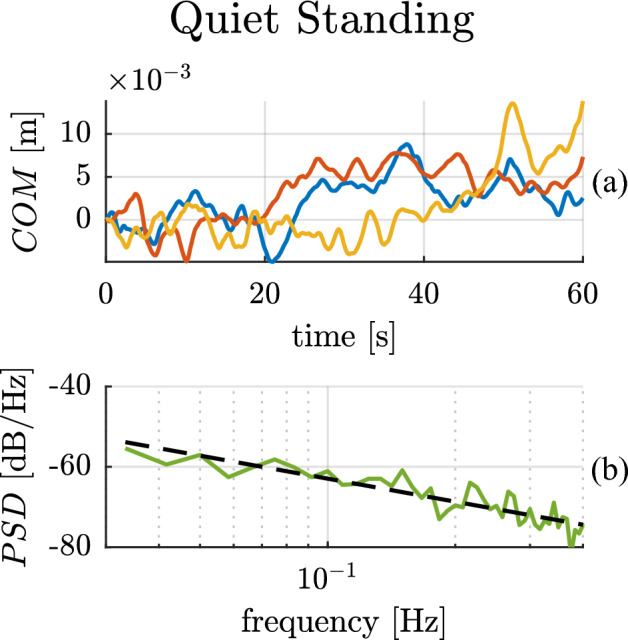
Quiet standing experimental results for a single subject. The top panel (**a**) shows the ‘x’ component of the center of mass trajectories over time for multiple trials. The bottom panel (**b**) presents the average Bode magnitude plot of the power spectral density of the ‘x’ component of the center of mass trajectories (solid green line), while the black dashed line presents the best-fit line with slope −19.05 dB/dec.

The experimental results for a single subject are presented in Fig. 4. The two panels show: (a) the time trajectories of the CoM position over the 3 trials, and (b) the related Bode magnitude plot of the PSD. The x-coordinate of the CoM is presented; the y-coordinate (omitted for clarity) exhibited similar behavior.

The CoM trajectory of the subject (Fig. 4.a) shows evidence of random fluctuations. The Bode magnitude plot of the PSD of these fluctuations follows a linear ($${R}_{PSD}^{2}=0.79)$$ slope of $$-19.05$$ dB/dec in ‘x’, with 95% CI $$[-22.09 -16.00]$$ dB/dec, and a slope of $$-18.27$$ dB/dec ($${R}_{PSD}^{2}=0.73)$$ in ‘y’ with 95% CI $$[-21.69-14.86]$$ dB/dec, over the low-frequency range (0.03–0.4 Hz) as shown in Fig. 4.b.

A similar behavior was observed across all subjects, with an average Bode magnitude slope of $-16.06$ dB/dec (${R}_{PSD}^{2}=0.70\pm 0.14)$ with 95% CI on both directions of $[-19.12 -13.00]$ dB/dec, displaying once again a flatter-than-Brownian slope. The “Supplementary Information” contains the best-fit PSD slopes, 95% confidence intervals, and coefficients of determination ${R}_{PSD}^{2}$ for all subjects.

### Descriptive model

The crank-turning experiments displayed the signature of a purely Brownian process, while the hand-posture experiments showed signs of variance growing linearly with time only up to a certain time point, after which the variance stopped increasing. These observations are consistent with our expectations: position variance must be bounded for postural tasks. However, it is possible that all three tasks share a common control architecture that produces a Brownian process, with an additional layer of control responsible for bounding the variance in postural tasks. Here, we develop a descriptive model assuming that a similar control structure underlies all three tasks.

**Figure 5:**
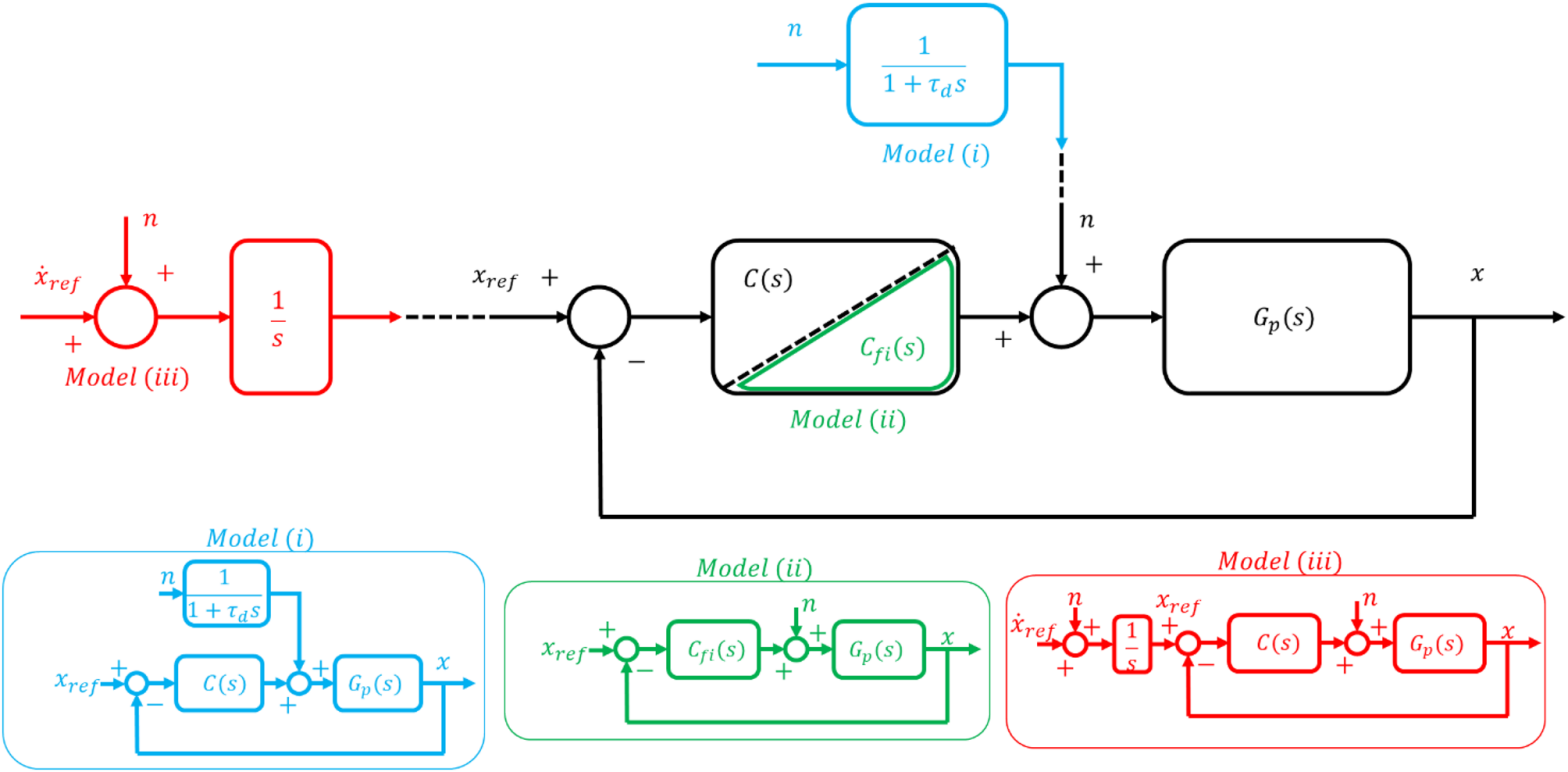
Block diagram of a closed-loop negative feedback control system with the three different ways to generate Brownian-like behavior in position: model ‘i’ (light blue) consists of an additive external stationary noise ‘$n$’ processed through a first-order low-pass filter (a ‘leaky integrator’); model ‘ii’ (green) requires a constrained design of the controller ‘${C}_{fi}(s)$’ such that the closed-loop dynamics exhibits a free integrator; model ‘iii’ (red) introduces a velocity level command ‘${\dot{x}}_{ref}$’ affected by stationary noise ‘$n$’ and integrates it to obtain the reference position command ‘${x}_{ref}$’. ‘${G}_{p}(s)$’ represents the open-loop dynamics of the physical system being controlled, whether it is the arm for the crank-turning and hand-posture tasks, or the full body in upright stance for the quiet standing task. ‘${G}_{p}(s)$’ receives joint torque commands as input, and outputs the position measurement of interest for the given task.

A general description of all three tasks requires a model with closed-loop negative feedback. In this case, Brownian behavior, in position, can emerge from only three sources: (i) an additive external disturbance as originally proposed by Peterka^23^; (ii) a free integrator in the closed-loop dynamics; and (iii) a forward-path (reference) velocity command, corrupted by stationary noise and integrated to produce a position reference command. Figure 5 provides a schematic representation of these three possibilities. A detailed description of all three models is presented in the “Supplementary Information”. In the following we assess their biological plausibility.

#### Additive external disturbance—model ‘i’

This model uses a linear time-invariant structure with $C(s)$ representing the control action and ${G}_{p}(s)$ the open-loop dynamics. The system is driven by a position reference command ${x}_{ref}$, while the controller output is corrupted by an additive noise signal $n$. To account for the boundedness of postural tasks (hand posture and quiet standing) the noise is modified by a first-order low-pass filter (a ‘leaky integrator’) added to the input of the physical system (e.g. muscle forces applied to the skeleton)^23^. The presence of the leaky integrator guarantees the emergence of the bounded postural behavior. However, this requires the process time-constant (${\tau }_{d}$ in Fig. 5) to be fast enough that the bound is reached in a finite time window as in typical observations (see experimental behavior of Fig. 3.a). At the same time, the process time-constant ${\tau }_{d}$ must be slow enough to reproduce the apparently unbounded variance of the crank-turning data in Fig. 2. Strictly Brownian behavior over the time window of our crank-turning observations (26 s) would require a time-constant of about 75 s, which seems implausibly long. Based on the data we observed, these two requirements appear to be incompatible. For a detailed presentation of model ‘i’, please refer to the “Supplementary Information”.

#### Free integrator in the closed-loop dynamics—model ‘ii’

The second model also exploits a position-feedback-loop control architecture. In this case, the system noise $n$ is stationary and directly added to the input of the open-loop dynamics ${G}_{p}(s)$. This model requires the implementation of a controller ${C}_{fi}(s)$ capable of producing a free-integrator $\left(\frac{1}{s}\right)$ behavior in the closed-loop dynamics: $\frac{{C}_{fi}\left(s\right){G}_{p}\left(s\right)}{1+{C}_{fi}\left(s\right){G}_{p}\left(s\right)}$. From a classical control perspective, this means placing a pole at the origin of the closed-loop transfer function and that requires complete knowledge of the system to be controlled (‘${G}_{p}(s)$’ in Fig. 5). In the context of standing balance, the extrinsic de-stabilizing gravitational load must be exactly balanced by the intrinsic neuro-muscular stiffness, a topic debated in the standing-balance literature, which makes this model less plausible. A free integrator would guarantee a strictly Brownian behavior in position, thus reproducing the crank turning data of (Fig. 2). To account for the postural data (Figs. 3 and 4), some process to limit position variance is required in addition. For a detailed presentation of model ‘ii’, please refer to the “Supplementary Information”.

#### Forward-path (reference) velocity command, corrupted by stationary noise—model ‘iii’

The third model uses velocity-based feedback control. The controller $C(s)$, the open-loop dynamics ${G}_{p}\left(s\right)$, and the additive input noise $n$ are not subject to particular constraints, as long as the feedback control loop exhibits stable close-loop dynamics. This model guarantees the emergence of Brownian postural behavior by means of a forward-path velocity command ‘${\dot{x}}_{ref}$’ corrupted by stationary noise ‘$n$’ (see Fig. 5). In this case a Brownian process is already embedded in the command signal ‘${x}_{ref}$’ and no particular knowledge of the control system ($C(s)$ or ${G}_{p}(s)$) is required. Similar to model ‘ii’ (the free integrator), some form of correcting action is required to prevent position variance from growing without bound during postural tasks.

#### Intermittent control

One biologically-plausible means of bounding variance is through intermittent control actions triggered by crossing a threshold. Intermittent control has been proposed frequently in the motor neuroscience literature^28–36^. Intermittency is characterized by an alternation between ‘do-something’ and ‘do-nothing’ regions. In control theory, this can be achieved by designing a controller that produces an output (‘do-something’) only when certain conditions e.g., measurement thresholds, are met; otherwise, the intermittent control output will be null (‘do-nothing’). The form of the control output as well as the type of triggering events are design parameters. In our specific case, the intermittent controller to bound the postural variance used the position $x$ (refer to Fig. 5) as the threshold variable and a gaussian-shaped control output as the intermittent action. Please refer to the “Supplementary Information” for details of the mathematical formulation.

**Figure 6:**
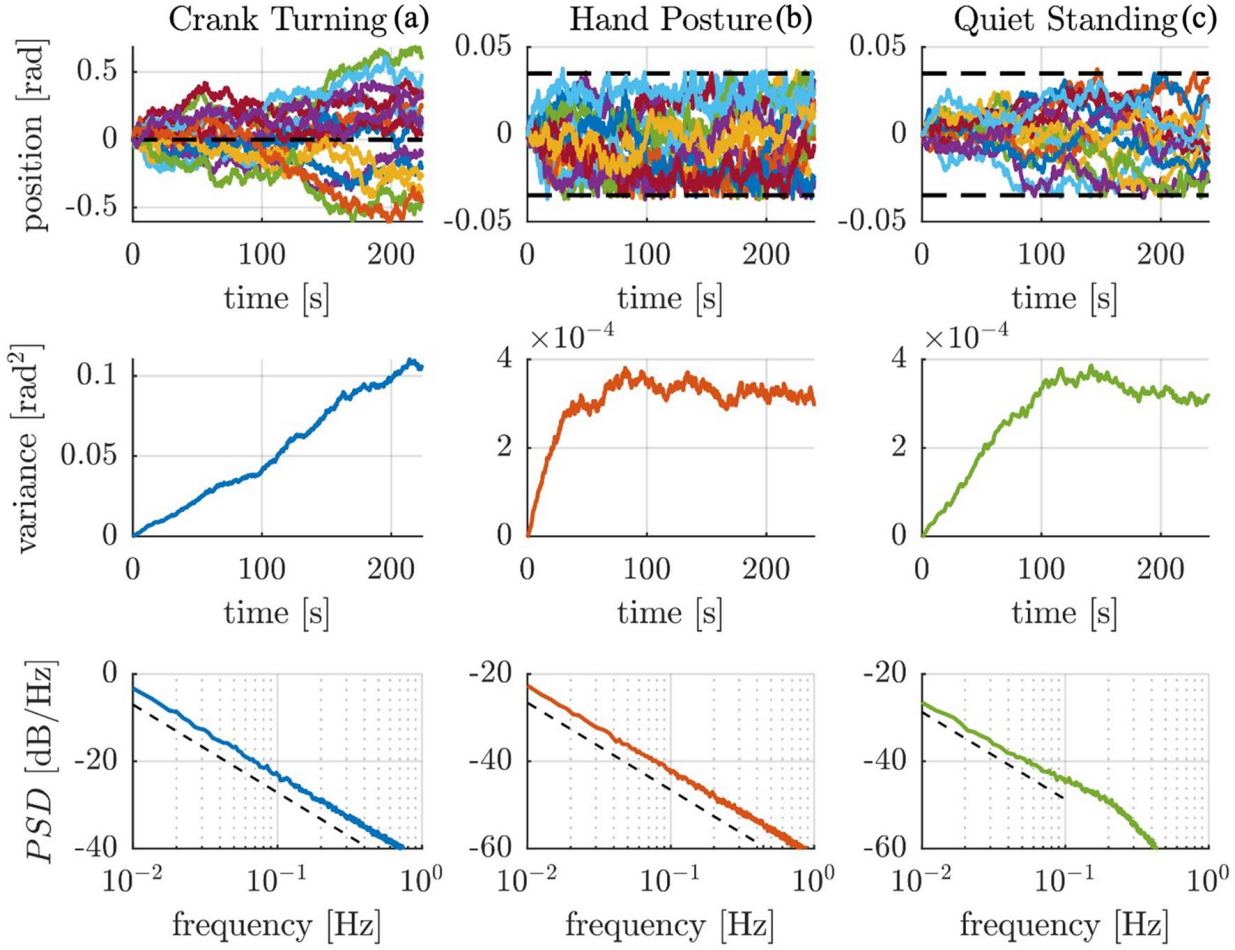
Simulation results of the proposed control model across three tasks: (**a**) crank turning, (**b**) hand posture, and (**c**) quiet standing. The top row of plots shows the time evolution (colored lines) of position relative to the expected nominal trajectory in the three cases; the black dashed line in the crank-turning trajectory plot represents the expected nominal trajectory for the target crank-turning speed, while the black dashed lines in the right two trajectory plots present the thresholds to trigger intermittent control action in the posture control tasks. The middle row of plots shows the variance over time across the ensemble. The bottom row presents the Bode magnitude plots of the average power spectral densities in the three different tasks. An additional -20 dB/dec dashed-black reference line is added to facilitate interpretation of the results.

Of the three proposed models, we report here the simulation results of model ‘iii’ with intermittent control, which was able to account for all the experimental evidence. The performance of the unsuccessful models is reported in the “Supplementary Information”. A total of 200 numerical simulations per condition were performed to assess the competence of the proposed model to reproduce the experimental observations. Figure 6 shows the model performance in the three different tasks: crank turning, hand posture, and quiet standing.

The model reproduced both the unbounded Brownian behavior during crank turning (Fig. 6a) as well as the bounded behavior during hand posture and quiet standing (Fig. 6b,c). The PSDs (Fig. 6) present a low-frequency (0.01–1 Hz for crank turning and hand posture, 0.01–0.1 Hz for quiet standing) Bode magnitude slope consistent with the experimental evidence: crank-turning: $-19.70$ dB/dec with 95% CI $[-19.78 -19.61]$ dB/dec; hand posture: $-19.42$ dB/dec with 95% CI $[-19.50 -19.34]$ dB/dec; and quiet-standing: $-17.94$ dB/dec with 95% CI $[-18.35-17.53]$ dB/dec.

## Discussion

### Experiments

The experimental PSDs in the crank-turning case show a slope remarkably close to $-20$ dB/dec (Fig. 2c). This behavior indicates a strictly Brownian process at low frequencies. In the hand-posture and quiet-standing cases, we observed a flattening of the PSD slope at low frequencies (Figs. 3c and 4b). At higher frequencies, the slope varied.

Reaching with the upper limb has been studied widely in motor neuroscience^37–41^. A kinematic constraint provides an intermediate stage between unconstrained (free) motion and interaction with complex dynamics. Circularly constrained motion, such as crank-turning, has been less widely studied^42–44^, despite being ubiquitous in everyday manipulation (e.g., turning a steering wheel or opening a door). In fact, opening a door was reported to be the most common activity of daily living^26^. The crank-turning task occupies a finite region of the arm’s workspace; as a result, the displacement of the hand relative to the thorax is bounded. However, the angular position of the crank is unbounded. Thus, the variance of angular position may grow without bound, and in fact it grew linearly (Fig. 2.2), indicating a Brownian process. It is worth emphasizing that this particular task allowed an unbounded task-space motion to be executed by motor actions in a finite joint space.

In the postural tasks, such as maintaining a static hand posture or quietly standing, the results also matched the predictions. In these cases, a Brownian-like behavior was still observed as a growing position variance until it reached a limit (Fig. 3b). This was confirmed by the position trajectories (Fig. 3a) where variance initially grew but was ultimately bounded. All subjects exhibited a transition between approximately linear growth and boundedness, but its onset varied among subjects, ranging from a few seconds to hundreds. Interestingly, a later onset of the bounded behavior corresponded to PSD slopes closer to an unbounded Brownian process ($-20$ dB/dec), while an earlier onset correlated with flatter PSD slopes. A possible explanation for this wide range of transition times could be individual differences in subjects’ sensorimotor performance as well as the level of concentration while performing the task.

#### Implication of Brownian processes in biology

A Brownian process—also known as a Wiener process or random walk—is a non-stationary process characterized by variance that grows linearly with time^19^. First discovered by botanist Robert Brown in 1828, it found a more formal definition in one of Einstein’s *annus mirabilis* papers in 1905^19^, and was rigorously described mathematically as a stochastic process by Norbert Wiener many years later^18^. Evidence of Brownian processes has been found in many fields: the behavior of particles in a stationary liquid, in chemistry^45^, electromagnetism^46^, fluid-dynamics^47^, and the trends of stock prices^48^. Our results provide evidence that it is a ubiquitous (and perhaps essential) feature of human motor behavior.

The existing literature has reported phenomena similar to Brownian motion, often termed “drift” or “sway”, in the CoP trajectory during quiet standing. The origin of this “drift” and the underlying human motor control mechanisms have been discussed at length. Collins and De Luca were the first to suggest the presence of “fractional Brownian” behavior in CoP postural sway, proposing an alternation between “do-nothing” (open-loop) and “do-something” (closed-loop) control actions to explain the observations^21^. Thereafter, several studies considered many aspects of this postural “sway”: its dependency on sensory information^22^, its possible decomposition into different motor control mechanisms^49^, the possibility of describing it with linear models^23^ or intermittent control^32,36^, the kind of stability necessary to explain it^33,34^, or the use of different mathematical methods to characterize it^24,25,50^. Similar studies were extended to other tasks involving balancing or pointing objects with the upper limbs^51,52^. In these cases, too, related work studied, for example, the influence of sensory information^53^ or the underlying control mechanisms^51^.

A fractional Brownian process is associated with chaotic dynamics and implies a nonlinear growth of variance with time, unlike the linear growth of variance that characterizes a Brownian process. Our results, however, indicate no need to postulate fractional behavior. In fact, we believe that the apparent fractional behavior reported in previous work^22,24,25^ may be a consequence of the inertial properties of the system and the bounded behavior during the postural tasks. A detailed example is presented in the “Supplementary Information”.

The results reported here establish that a Brownian process is a good description of intrinsic position variability. However, some additional process must act to limit position variance in postural tasks. One possible explanation can be found in the theory of intermittent feedback control. Intermittency in human motor actions has been reported since Woodworth in 1899^54^, and fundamentally arises – in its simplest form – by alternating a “do-nothing” region for small feedback errors within which there is no control action, with “do-something” regions to correct for larger feedback errors. Several studies have supported intermittent control in human motor behavior^21,29,32–35,51,55^. Most of the cited studies focused on the presence of intermittent control during upright posture or balancing tasks, showing promising results. Intermittency is a non-linear process i.e.; the alternating of action and no-action will lead to discontinuities not accountable by linear models. A possible linear alternative to intermittency was proposed for upright posture by Peterka^23^: in his work, he showed how postural sway could be reproduced using a ‘leaky integrator’ without any need for intermittency. His proposal was tested in model ‘i’. The analysis of this model is discussed in further detail in the following section and in the “Supplementary Information”.

### Models

The proposed descriptive model (model ‘iii’ of Fig. 5) exhibits Brownian behavior by means of stationary noise added to a reference (forward-path) velocity command. This is equivalent to including a “free integrator” upstream of the interactive dynamics in a Norton equivalent network^56^. To bound the variance in postural tasks, an intermittent controller was included in the forward path where the “do-something” region outside the feedback-error thresholds generated a corrective action as a velocity command. For simplicity, linear representations of the open-loop system dynamics ${G}_{p}\left(s\right)$ and compensator $C\left(s\right)$ were assumed but that is not essential. We emphasize that this work identified a feature in one of the lowest levels of a complex control system. It is silent about the form of the higher-level controller as long as it presents intermittent feedforward action as velocity commands. Interestingly, this is consistent with recent robotic implementations^57^. Figure 6 shows that this model can reproduce all of our experimental observations: crank turning, hand posture, and quiet standing. It is also important to emphasize that the behavior observed in model ‘iii’ can only be achieved with a reference velocity command and not by higher order derivatives. For example, acceleration or jerk reference commands would require double or triple integration to get the position signal. White noise going through multiple integrators (> 1) would not yield a Brownian process, but higher-order processes with PSD characterized by a slope of $-20\cdot n$ dB/dec, where $n$ is the order of integration. The resulting system would not be marginally stable i.e., presenting a random walk and linearly growing variance, but unstable.

Model ‘iii’ is not the only possible way to generate a Brownian process in position: there are two alternatives. The first alternative (model ‘i’) was inspired by Peterka^23^ who showed that the phenomena reported by Collins and De Luca^21^ could be reproduced by a continuous linear time-invariant controller with a low-pass-filtered stationary noise disturbance, with no need for intermittent ‘open-loop’ and ‘closed-loop’ action. Peterka’s model requires an appropriate tuning of the time constant (‘${\tau }_{d}$’ of Fig. 5) to produce the desired bounded behavior in postural tasks. This time constant ranged between 10 and 120 s in Peterka’s work, which appears difficult to justify for human motor control. In our simulations, we could reproduce the bounded behavior of hand posture and quiet standing with the time constant ranging from 20 to 160 s. However, in order to reproduce the crank turning behavior we had to use a time constant of at least 78 s and as much as 240 s. As this model requires such a wide range of time constants to account for all of the experimental evidence, we believe that it is not the most suitable to describe all three tasks investigated in this work. Results highlighting the limitation of model ‘i’ are reported in the “Supplementary Information”.

The second alternative model (‘ii’) requires the system’s closed-loop transfer function to exhibit exactly one pole at zero to produce a Brownian process in position. In principle, this could be achieved by a controller that compensates exactly for gravitational destabilization during upright posture or by nullifying arm stiffness during gravity-neutral hand posture. Whether intrinsic joint stiffness is sufficient to stabilize standing posture has been debated in the balance literature^58,59^, and exactly balancing extrinsic and intrinsic loads seems challenging, to say the least. However, to cancel gravitational stiffness requires less joint stiffness than to stabilize posture and it might be interesting to re-consider this matter in light of this reduced requirement. To maintain a gravity-neutral posture of the hand would require imposing a negative stiffness to compensate for intrinsic muscle impedance, which is positive^60,61^. Feedback processes (e.g. via spinal reflexes) could generate ‘negative stiffness’, and apparently do so in some circumstances ^62,63^. In this second alternative model, an intermittent control action would suffice to bound the variance in postural tasks. Nonetheless, this model fails to reproduce the unbounded task i.e., crank-turning. Having a net stiffness equivalent to zero is equivalent of having a velocity tracking controller with no integral action (stiffness) which will cause a steady-state error in the velocity tracking performance, and thus position—integral of velocity—will grow with a different slope (rate) compared to the required reference. This is not what we observed experimentally i.e., the measured position grew with the same slope (rate) of the reference target velocity. Results highlighting the limitation of model ‘ii’ are reported in the “Supplementary Information”.

In all the considered models, the noise has been modeled as an additive quantity. However, this does not preclude the presence or possibility of signal-dependent noise. As highlighted by Harris and Wolpert, signal-dependent noise plays an important role during motor planning^17^. In the simulated tasks, the noise was added to either a zero (postural task) or constant (crank turning) reference velocity. Therefore, even if present, the signal-dependent noise would not produce changes that would affect the emergence of the Brownian process. The assumption to use stationary white noise sources is a first-degree approximation that allows for a wide set of mathematical manipulation tools that provide intuitive insight into the underlying mechanisms. Moreover, according to Faisal et al., it is also the ‘best’ assumption in data-limited contexts for human neuromotor control studies^16^.

Of course, it is possible that different control architectures underlie different motor tasks, and one may argue against constraining ourselves to a model that can describe all of the experimental observations in the three different tasks. However, given the pervasiveness of drift, Brownian behavior appears to be a fundamental feature of neuromotor control, and it is plausible that this Brownian process is a manifestation of some common control architecture shared across the various motor tasks i.e., velocity-level planning.

Which mechanical variables best correlate with neural activity is controversial, and irrelevant in some schools of thought. This is probably because many factors are important for successful motor behaviors. Limb velocity, displacement, force, and force rate initially presented plausible correlation values^64^. Knowledge of neural encoding is still evolving. More recently, another perspective has emerged, suggesting that muscle activation may result from the collective activation of many neurons which generate rotational dynamics to produce a learned feedforward action that plays out over a finite duration^65,66^. However, our discussion of velocity commands does not preclude its relevance to such a representation.

Apart from successful implementation in brain-machine interfaces, the importance of velocity commands in motor tasks has been highlighted in numerous other studies. Evidence of velocity-level planning is found in the work of Atkeson and Hollerbach^67^ on spontaneous reaching tasks, where they observed highly-stereotyped speed profiles despite substantial variation of hand paths. Krebs et al. reported evidence of stereotyped upper-limb speed profiles in the earliest spontaneous movements of recovering stroke-survivors^68^. In a study of re-targeting reaching movements, Flash and Henis found results consistent with stereotyped hand speed profiles^69^. Evidence of velocity commands has also been found in other neural circuits: Robinson, studying saccadic eye movements, proposed that premotor neurons encoded movement velocity and duration^70,71^; Rodman and Albright reported velocity coding in the middle temporal visual area cells of macaque^72^; Keshavarzi et al. recently reported that retrosplenial cortical neurons reliably track direction and speed of head turning^73^. All these studies point to velocity as a significant variable in neuromotor control, perhaps for the same reasons that velocity commands are useful in engineering applications.

The observations of Brownian processes in the human motor tasks presented in this work further support the idea of velocity-level planning for movement control: a model with forward-path velocity commands corrupted by stationary noise, and intermittent control action during postural tasks, is capable of reproducing all of our experimental observations. If the brain interprets descending (forward-path) motor commands in terms of velocity^6–8^, then because of noise, the resulting position must express a Brownian behavior, at least before intermittent correction—just as we observed. To the best of our knowledge, this model, together with the experimental evidence presented, provides behavioral support for the hypothesis that some aspects of neural activity can be interpreted as velocity commands.

## Material and methods

### Subjects

Two different human subject experiments were conducted at the Massachusetts Institute of Technology: crank turning (10 right-handed college-aged male subjects), and static hand posture control (4 males and 6 females, ages 23–36). The informed consent and experimental protocols were reviewed and approved by the Institutional Review Board for the Massachusetts Institute of Technology. We confirm that all research was performed in accordance with relevant guidelines/regulations and informed consent was obtained from all participants and/or their legal guardians before the experiment. The quiet standing data came from a public data set of experiments conducted by Santos et al. (2017) and consisted of data from 49 subjects^27^; the data for the 26 young unimpaired subjects in the study (15 males and 11 females, ages 18–40) were used in this paper.

### Crank turning experiments

Subjects turned a crank mounted on a high-precision incremental optical encoder/interpolator set (Gurley Precision Instruments encoder #8335-11250-CBQA, interpolator #HR2-80QA-BRD) with a resolution of 0.0004 degrees per count.

During the experiment, the subject’s arm was occluded from view by a wooden structure, which did not limit the range of motion. The upper arm was suspended by a canvas sling connected to the ceiling using a steel cable; the upper arm and lower arm were in the plane of the crank. The subject sat in a chair with a rigid back, while the shoulder was restrained by a harness attached to the back of the chair. The subject was positioned such that the crank, with radius 10.29 cm, was well within the workspace of the arm. Figure 1 shows a graphical representation of the experimental setup.

The encoder, sampling at 200 Hz, was connected to a set of counters and to the computer via digital I/O. The visual display, also generated by the computer, was on a 17-inch monitor (311 × 238 mm, resolution 1280 × 1024, 76 Hz) which was mounted approximately 75 cm from the subject’s eyes. The experiment was divided into two unequal sections: 2 blocks of trials at the subject’s preferred or ‘comfortable’ speed and 6 blocks of trials at a visually-instructed speed.

The experimental paradigm was reported by Hermus et al.^74^. The data is available at^75^. The results presented herein only include analysis of the slow, clockwise-turning condition. However, details of the entire experimental procedure are included for completeness.

At the start of the experiment, subjects performed 20 trials at their preferred speed, 10 trials in the clockwise direction (CW) and 10 in the counterclockwise direction (CCW); both conditions were blocked, in random sequence for each subject; each trial lasted 8 s. Subjects were not provided with any visual feedback during these trials. Thereafter, subjects performed 6 blocks of 30 trials, each with visual specification of 1 of 3 target speeds, in either CW or CCW directions. The order of the speed and direction blocks was pseudo-randomized across subjects. The three speeds were selected to cover a significant range: 0.075 rev/s was extremely slow (required over 13 s per revolution), 0.5 rev/s was close to subjects’ preferred speed, and 2.0 rev/s was close to the fastest speed that subjects could turn the crank. Visual feedback on the monitor displayed the target speed, as well as the subject’s real-time hand speed; the horizontal axis was time, and the vertical axis was speed. Target speed was displayed as a continuous horizontal line in the middle of the screen. The width of the screen corresponded to the time of the trial. 7 catch trials without visual feedback were included in each block, leaving 23 trials per block with visual feedback.

In the slow-speed conditions, each trial lasted 45 s. This yielded about 3.4 turns of the crank for the slow condition. The duration of the slow-speed trials was chosen as a compromise between acquiring adequate data and avoiding subject fatigue. The first and last cycle were removed to eliminate transients. This yielded a 26-s trial duration for analysis. The first 2 trials of each block were considered to be familiarization trials and were thus removed together with the 7 catch trials without visual feedback, leaving 21 trials per subject for analysis.

### Hand posture experiments

In the hand posture experiment, subjects were asked to hold the handle of an InMotion2 robot (Interactive Motion Technologies Inc.) and maintain a static hand posture at the center of a circle of radius 2.5 cm. The InMotion2 is a highly back-drivable two-link manipulandum. In the experiments, the robot served only as a position measurement device as the actuators were open-circuited. InMotion joint positions were measured by a 16-bit/rev encoder at ~ 350 Hz using a Compact RIO real-time processor. Data were interpolated to a sampling rate of 200 Hz.

The experimental setup was the same as the previous crank experiment except that the crank was replaced with the InMotion2 robot, vision of the hand was not occluded, and the visual feedback was changed to show the fixed target position (given by a circle of radius 2.5 cm) and the subject’s actual hand position (given by a dot of radius 1 cm). Figure 1 shows the experimental configuration. Each trial lasted 4 min, and was repeated 10 times for each subject, with breaks between trials.

### Quiet standing experiments

The quiet standing experiments were conducted by Santos et al. (2017) in the Laboratory of Biomechanics and Motor Control at the Federal University of ABC, Brazil^27^. Subjects were asked to stand barefoot, as still as possible, with their arms by their sides, under four conditions: on a rigid surface with eyes open, on a rigid surface with eyes closed, on a compliant surface (foam blocks) with eyes open, and on a compliant surface with eyes closed. Trials lasted 60 s and were performed 3 times per condition in random order. 3D kinematics were measured using a motion capture system, with data sampled at 100 Hz; post-processing was performed to estimate the center of mass (CoM) trajectory in Cartesian coordinates, as well as joint angles. Figure 1 shows the experimental configuration. Further experimental details can be found in the article by Santos et al. (2017)^27^.

The results presented herein include data for the first condition (eyes open, rigid surface), and for the 26 young unimpaired subjects. The estimated CoM trajectories included in the public data set were used in the data analysis. Note that the chosen (x,y,z) coordinate directions (see Fig. 1) are different from those used in the public data set by Santos et al. This was done to have the (x,y) coordinates in the horizontal plane for both quiet standing and hand posture.

### Data analysis

The data—crank angular position, hand position (x,y), and CoM position (x,y)—were processed using MATLAB (version 2022a). Three different quantities were analyzed: (i) the time trajectory of the position data over each trial, (ii) their variance-over-time computed across the ensemble, and (iii) their average power spectral density.

The time trajectory was directly extracted from each trial of the collected data. For all research studies (crank turning, hand posture, and quiet standing), data from all trials were aligned such that the initial data point was taken to be at the origin, namely $0 rad$ for the crank turning angle, and $\left(\mathrm{0,0}\right) m$ for the hand and CoM Cartesian coordinates.

The variance across the ensemble was computed at each time step (${t}_{s}=0.005 s$) using the time trajectories (aligned at the initial data point) with the following equation:

$$\sigma^{2} \left( t \right) = \frac{1}{N - 1}\mathop \sum \limits_{i = 1}^{N} \left( {x\left( {i,t} \right) - \frac{1}{N}\mathop \sum \limits_{j = 1}^{N} x\left( {j,t} \right)} \right)^{2}$$

where $i$ and $j$ represent the trial indices ranging from 1 to N (number of trials), and $x(i,t)$ represents the value of the considered variable at time $t$ during the $i$-th trial.

The power spectral densities (PSDs) were computed for each trial with Welch’s method^76^ using each entire trial as one window of data (in order to obtain spectral estimates down to the lowest possible frequency) and averaging across trials. The data were pre-processed by removing the average crank angle trajectory from the crank angle data, and removing the mean position over each trial for the hand position and CoM position data. The PSDs were post-processed to remove the lowest-three frequency points, as these depended sensitively on the data processing technique used (see the “Supplementary Information” for details of the pre- and post-processing decisions).

### Modeling

**Figure 7:**
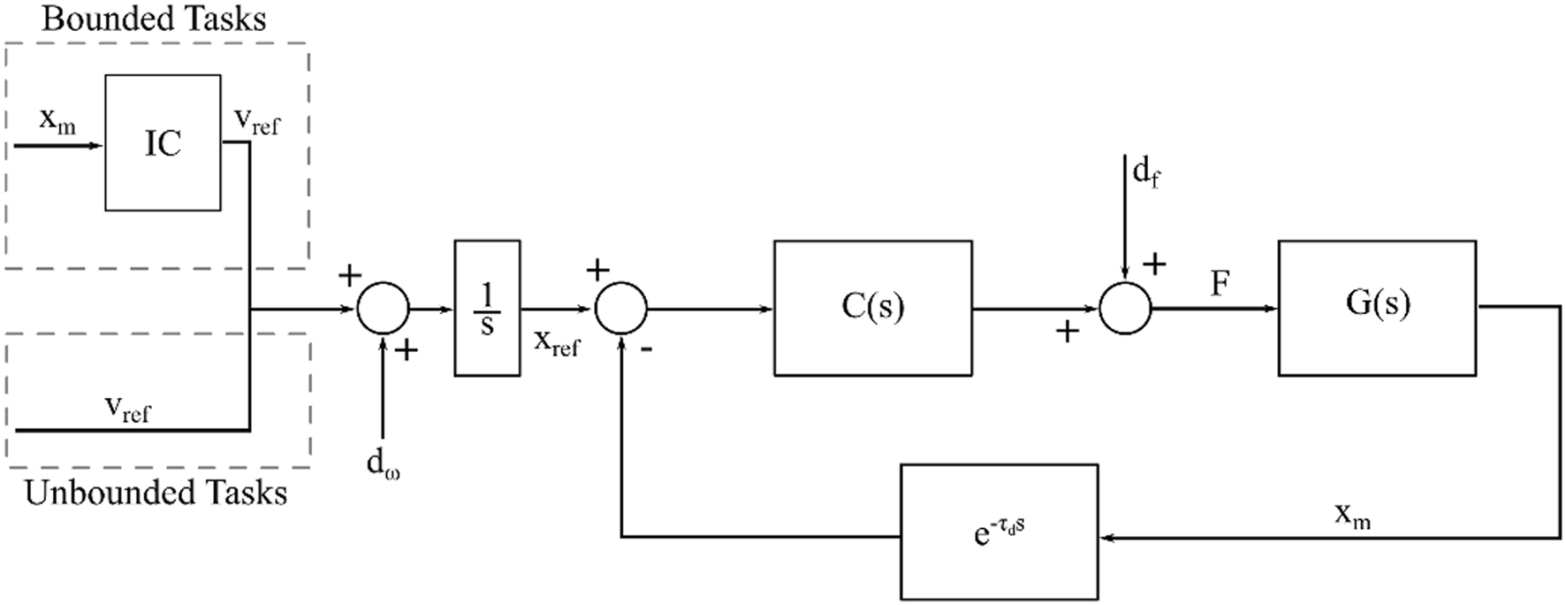
Block diagram of the modelled control scheme. G(s) represents the open-loop system dynamics, where s is the Laplace variable; C(s) is the closed-loop control action; IC represents the intermittent controller necessary to bound position variance; ${d}_{f}$ and ${d}_{\omega }$ represent additive noise on the control action (force/torque) and on the reference velocity command, respectively; ${\tau }_{d}$ represents sensor delay; F is the control action; ${x}_{ref}$ is the reference position; and ${v}_{ref}$ is the reference speed.

The descriptive mathematical model, represented in the block diagram of Fig. 7, was implemented in Simulink/MATLAB (v 2022a) to simulate the behavior of the three different motor tasks: crank turning, hand posture, and quiet standing. For each of these cases, a single-degree-of-freedom model was considered for simplicity.

Additive disturbance sources were included at the reference motion command and at the control action, denoted as ${d}_{\omega }$ and ${d}_{f}$ respectively. Both disturbances were modeled as white noise. A constant delay of ${\tau }_{d}=100 ms$ was included in the feedback path to account for feedback delays in the human sensorimotor system. The results were robust to decreasing the delay ${\tau }_{d}$; if ${\tau }_{d}$ was set to zero the same results were observed.

An idealized transfer function for the linearized open-loop system $G(s)$, where $s$ is the Laplace variable, was designed to represent two configurations: a gravity-neutral arm ${G}_{arm}(s)$ or upright stance ${G}_{stance}(s)$:

- $${G}_{arm}\left(s\right)=\frac{1}{m{L}^{2}{s}^{2}+\beta s}$$
- $${G}_{stance}\left(s\right)=\frac{1}{m{L}^{2}{s}^{2}+\beta s-mgL}$$

In the gravity-neutral-arm case, the system dynamics ${G}_{arm}(s)$ represents a marginally stable 2^nd^ order linear-time-invariant (LTI) system, with moment-of-inertia $m{L}^{2}$ and damping factor $\beta$, where $m$ is the total mass of the arm, and $L$ is the distance between the shoulder joint and the center of mass (CoM) of the arm. The distributed moment of inertia was neglected.

In the quiet-standing case, the system dynamics ${G}_{stance}(s)$ represents an unstable 2^nd^-order LTI system i.e., an inverted pendulum, with moment of inertia $m{L}^{2}$, damping factor $\beta$, and destabilizing gravitational stiffness $mgL$, where $m$ is the total mass of a human body, $L$ is the distance between the ankle joint and center of mass (CoM) of the whole body, and $g$ is the gravitational acceleration ($9.81 m/{s}^{2}$). Again, the distributed moment of inertia was neglected.

The closed-loop dynamics controller $C(s)$ was designed to guarantee asymptotic stability of the closed-loop transfer function. Specifically,

- For the gravity-neutral arm, we set $C\left(s\right)={k}_{c}>0$;
- For upright posture, we set $C\left(s\right)={k}_{c}>mgL$.

In static hand posture and quiet stance, an additional intermittent controller (IC) was introduced to bound the otherwise unbounded Brownian behavior. The chosen intermittent control performed no control action within the “do-nothing” region, while it generated a Gaussian velocity profile whenever the actual position measurement (${x}_{m}$) was outside the threshold of the “do-nothing” region. From a mathematical point of view, this was represented by the following control law:

$$v_{ref} \left( t \right) = { }\left\{ {\begin{array}{*{20}l} 0 \hfill & { if\quad - x_{th} \le x_{m} \left( t \right) \le + x_{th} } \hfill \\ {sgn\left( {x_{m} \left( t \right)} \right) \cdot v_{max} e^{{ - \frac{{\left( {t - t_{0} } \right)^{2} }}{{2c^{2} }}}} } \hfill & { if\quad x_{m} \left( t \right)\left\langle { - x_{th} \vee x_{m} \left( t \right)} \right\rangle + x_{th} } \hfill \\ \end{array} } \right.$$

where ${v}_{max}$ represents the peak velocity of the control command, ${t}_{0}$ is the timing offset, and $c$ is the standard deviation of the Gaussian profile, and $sgn\left({x}_{m}\left(t\right)\right)$ is the sign of the measured position. This intermittent control was based on the theory of dynamic motor primitives, and velocity-coded submovements specifically^77^. However, other control architectures that include intermittency, with a “do-nothing” region and a “do-something” region, would produce equivalent results as long as the “do-something” region included a sufficient corrective action to push the controlled system back into the “do-nothing” region.

200 simulations of this model were performed for each of the three motion tasks. The noise disturbance profiles were generated with pseudo-random white-noise sequences, initialized with different seeds to guarantee independence between the different simulations. Table 1 summarizes the values of the parameters used for the simulations. In each motor task, the position trajectory over time was sampled at a frequency of 100 Hz and then processed using the same data analysis methodology adopted for the experimental data.

**Table 1:** Parameters used in the simulations of the two cases considered: upper-extremity gravity-neutral arm posture and upright stance.

Parameter	Upper-extremity gravity-neutral arm posture	Upright standing balance
$m$	1.5 kg	75 kg
$\beta$	30 Nms/rad	226 Nms/rad
$L$	0.35 m	1 m
${k}_{c}$	65 Nm/rad	883 Nm/rad

### Supplementary Information

Supplementary Information.

## Data Availability

All data are available in the main text or the supplementary information. The MATLAB code and the experimental data are available at the following link: https://github.com/jameshermus/brownianProcess/tree/main.
