# Supplementary material for: Brownian processes in human motor control support descending neural velocity commands
Source: Sci Rep. 2024 Apr 9;14:8341. doi: 10.1038/s41598-024-58380-5 (PMC11004188; doi:10.1038/s41598-024-58380-5)
Supplement: Supplementary file 1 — Supplementary Information. [file 41598_2024_58380_MOESM1_ESM.docx]

**Supplementary Information**

**Spectral Analysis**

Power spectral densities (PSDs) were computed for each data set using Welch’s method [75]. Various data pre-processing techniques were compared with the goal of reducing any numerical artifacts in the computed PSDs. Three different detrending approaches were considered: (I) no detrending, (II) subtraction of the mean, and (III) linear detrending. Furthermore, two windows were tested on the data: (I) rectangular window, and (II) Hanning window. Fig S1 shows PSDs computed using different detrending and windowing approaches for one subject performing the hand posture task. The different approaches mainly affected the three lowest-frequency data points, which were therefore removed from the reported PSDs. The window shape mainly affected the total signal power, without significantly affecting the shape of the spectrum. This can be explained by the fact that the Hanning window is used to taper the ends of the data window, effectively reducing signal amplitude. The Hanning window is typically used to obtain cleaner high frequency spectral estimates, reducing side-lobe artifacts that may be produced by other windowing methods, including the rectangular window. Since we were interested in the low-frequency spectrum, which is largely unaffected by the choice of the window, the final PSDs were computed using the rectangular window as it involved minimal processing of the data.


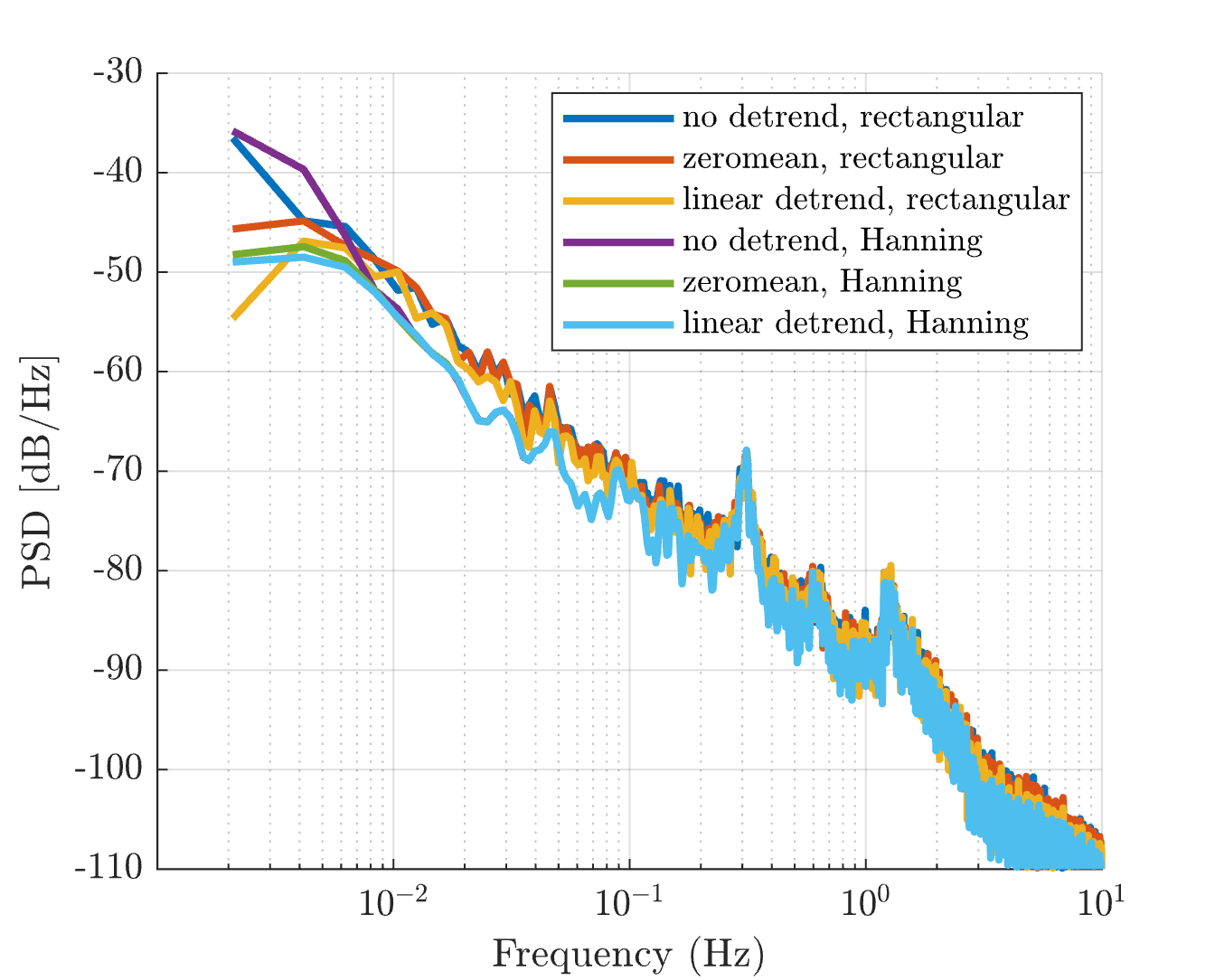


**Fig S1.** **Power spectral densities for a single subject performing the hand posture experiment.** They were computed using six different pre-processing techniques: no detrending with rectangular window (blue), zero-mean with rectangular window (red), linear detrend with rectangular window (yellow), no detrending with Hanning window (purple), zero-mean with Hanning window (green), linear detrend with rectangular window (light blue). The detrending process mainly affects the lowest-frequency data points, while the window shape affects total signal power.

For the crank-turning task, which involved displacements due to voluntary movement of the hand around the crank, the crank-angle data were detrended by removing the average trajectory over the 21 trials in order to focus the spectral analysis on the random fluctuations around the average trajectory.

**Alternative Models**

Two alternative models were investigated to describe a Brownian process in position emerging from a closed-loop negative feedback control system. The first was based on work proposed by Peterka [23], where stationary noise is filtered through a low-pass filter $\left( \frac{1}{1+\tau_{d}s} \right)$ with an extremely low cut-off frequency, a so-called ‘leaky integrator’, and injected as a disturbance into the feedback control loop (please refer to Figure 5 of the main document). Stationary noise filtered through a ‘leaky integrator’ generates a Brownian-like behavior that is bounded, with variance growing linearly over time until the low cut-off frequency of the filter is reached. Such a model reproduces the Brownian-like behavior observed in postural tasks, such as hand posture control or quiet standing, in which the variance is bounded. However, in order to reproduce the crank turning behavior we had to use a time constant of at least 78 s and as much as 240 s.

Figure S2 reports the result of 200 simulations performed using this first alternative model and considering a series of 5 different time constants, $\tau_{d}=\left[ 20 40 80 160 240 \right]ms$. For the simulation, the plant was modeled using the ‘upper-extremity gravity-neutral arm posture’ parameters presented in Table 1. The results highlight how model ‘i’, in order to reproduce the crank turning behavior i.e., linearly and unbounded growing variance, requires very large time constants (up to 240 s). On the other hand, it needs values as low as 20 ms to reproduce the bounded growing variance of postural task. As this model requires such a wide range of time constants to account for all of the experimental evidence, we believe that it is not the most suitable to describe all three tasks investigated in this work.

**Fig S2.** **Alternative model ‘i’ crank turning behavior.** The top panel shows the time evolution of the detrended angular position. The middle panel shows the normalized^[[1]](#footnote-1)^ variance over time across the different time delays. The bottom panel presents the Bode magnitude plots of the average power spectral densities for the five different time delays. An additional -20 dB/dec dashed-black reference line is added to facilitate interpretation of the results. Higher time constants are represented by darker lines.

A second alternative model describes the Brownian behavior in position as emerging from the closed-loop dynamics. In this case, the closed-loop transfer function requires exactly one pole at the complex-plane origin i.e., a free integrator. The controller required to produce this behavior differs depending on the open-loop dynamics G(s) of the system being considered, which can be:

1. unstable (at least one pole with a positive real part);
2. asymptotically stable (all poles with negative real parts);
3. marginally/neutrally stable (one pole at the origin).

For cases (I) and (II), the controller $C(s)$ was designed to cancel all unstable poles, and place one pole at the origin. In case (III), the open-loop system already presents a pole at the origin, so the controller $C(s)$ was designed such that the pole at the origin was not shifted. The following presents three different 2^nd^ order LTI systems e.g., mass-spring-damper systems, with $G(s)$ in each of the three previously mentioned cases, and the corresponding controller $C\left( s \right)$ required to place a single closed-loop pole at the origin:

1. If $G\left( s \right)=\frac{1}{ms^{2}+\beta s-k}$ , then $C\left( s \right)=k_{c}=k$
2. If $G\left( s \right)=\frac{1}{ms^{2}+\beta s+k}$ , then $C\left( s \right)= k_{c}=-k$
3. If $G\left( s \right)=\frac{1}{ms^{2}+\beta s}$ , then $C\left( s \right)=k_{c}=0$

The 2^nd^ alternative model, with the three variations described above to produce a free integrator, would guarantee the appearance of an unbounded Brownian behavior in position. To bound the Brownian behavior in postural tasks (hand posture and quiet standing), an intermittent controller was designed such that the control actions producing the free integrator were augmented to limit the position variance. In the postural tasks, the condition was a threshold in the position error: when the position error was within the thresholds, the closed-loop dynamics maintained a pole at the origin, while when the error exceeded the thresholds, the closed-loop dynamics exhibited an asymptotically stable behavior. Fig S3 presents a possible intermittent control action $C(s)$ for the three considered open-loop dynamics: unstable, asymptotically stable, and marginally stable.


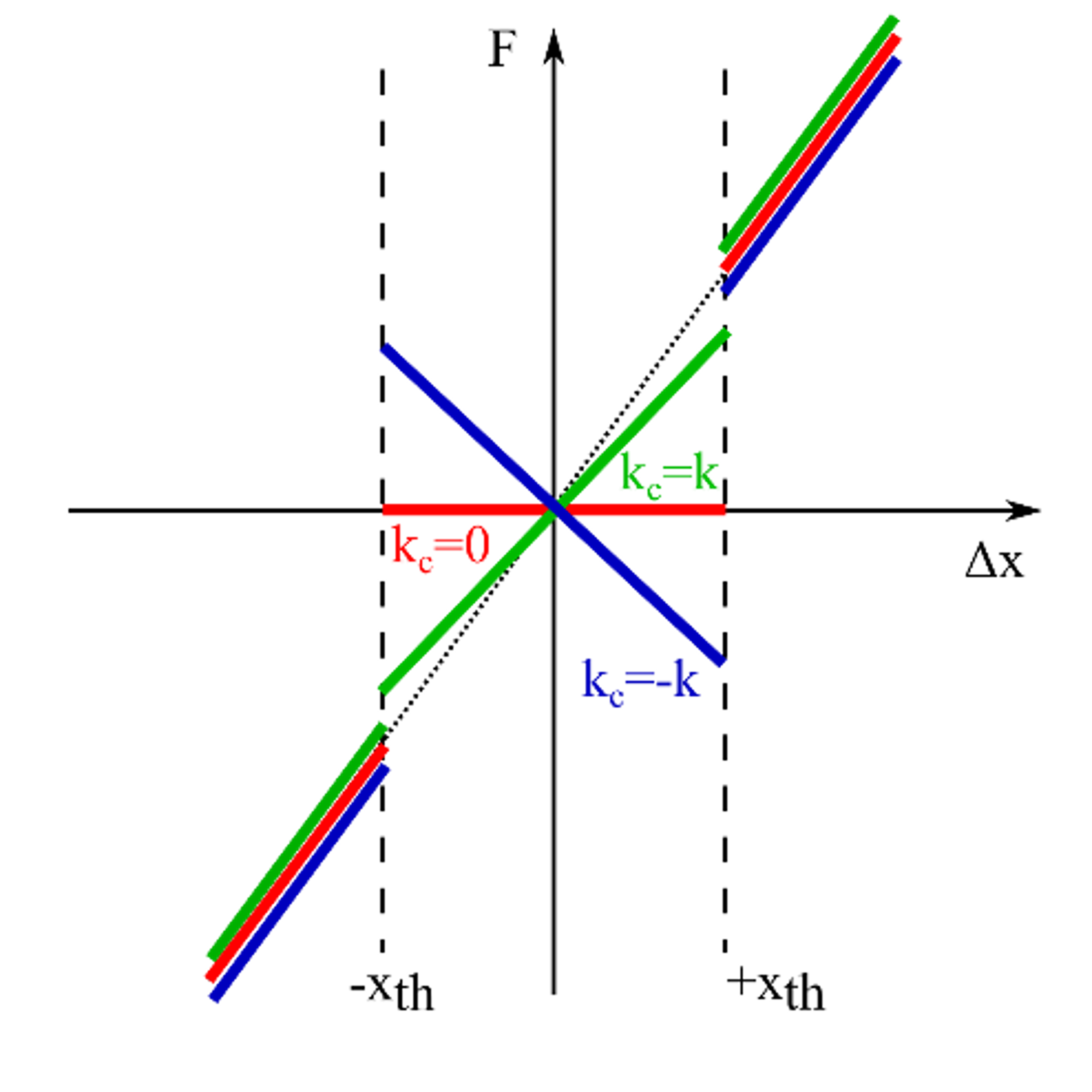


**Fig S3.** **Intermittent control action based on position thresholds** $\boldsymbol{\pm}\boldsymbol{x}_{\boldsymbol{th}}$ (black dashed lines). It was designed to guarantee a free integrator in the closed-loop dynamics when the error was within the thresholds for three different open-loop dynamics: unstable (green solid line), marginally stable (red line), and asymptotically stable (blue). Outside the thresholds the controller guaranteed an asymptotically stable behavior.

However, this model also fails to reproduce the crank-turning case. In fact, a net stiffness equivalent to zero leads to no integral action (stiffness) in a velocity-controlled system which will cause a steady-state error in the velocity tracking performance. This will cause the position – integral of velocity – to grow with a different slope (rate) compared to the required reference. This is not what was observed experimentally i.e., the variance grew but no steady-state velocity error was measured.

Figure S4 reports the result of 200 simulations performed using this second alternative model for the crank turning case. For the simulation, the plant was modeled using the ‘upper-extremity gravity-neutral arm posture’ parameters presented in Table 1. The top panel of Figure S4 shows the emergence of the growing position error i.e., the constant velocity error.

**Fig S4.** **Alternative model ‘ii’ crank turning behavior.** The top panel shows in blue the time evolution of the angular position, while - with a dashed black line – it presents the reference angular trajectory. The middle panel shows the variance over time. The bottom panel presents the Bode magnitude plots of the average power spectral density. An additional -20 dB/dec dashed-black reference line is added to facilitate interpretation of the results.

**Stabilogram Diffusion Analysis**

Stabilogram diffusion analysis was introduced by Collins and De Luca to study the fluctuations observed in the trajectory of the center of pressure (CoP) position during quiet stance [21]. It is a time-series analysis tool borrowed from statistical mechanics that involves computing the mean-squared displacement (MSD) of a signal for different time intervals to analyze the nature of the fluctuations present in the time series.

Consider a signal sequence $x\left( k \right)$ with $k=1, \ldots, N$, and let $\Delta t$ denote the span interval between two points of the $x(k$) data sequence. The mean-squared displacement can be computed as:

$$\Delta r^{2}\left( \Delta t \right)=\frac{\sum_{k=1}^{N-\Delta t} \left( x\left( k+\Delta t \right)-x\left( k \right) \right)^{2}}{N-\Delta t}$$

The mean-squared displacement can then be plotted with respect to different span intervals $\Delta t$ to produce a diffusion plot. The evolution of $\Delta r^{2}$ provides information on the fractality of the analyzed time series. In fact, it is known that a fractional random walk process is characterized by the following relationship between the mean-squared displacement and time:

$$\Delta r^{2}\sim{\Delta t}^{2H}$$

where the scaling exponent $H$ denotes the Hurst coefficient, and can be obtained from a log-log plot of $\Delta r^{2}$ with respect to $\Delta t$. When the slope 2H is exactly equal to 1, corresponding to $H=0.5$, the behavior follows a classical (purely) Brownian process, with variance that grows linearly with time. Instead, if $H\neq0.5$, the process is classified as a fractal or “fractional Brownian” process, which can be persistent ($H>0.5\boldsymbol{)}$ or anti-persistent $(H<0.5\boldsymbol{)}$ [21].

Collins and De Luca observed that their diffusion plots exhibited two distinct regions with different slopes: what they referred to as a “short-term” region and a “long-term” region. The slopes measured in the log-log diffusion plots suggested the presence of “fractional Brownian” processes in both regimes, with persistent behavior in the short term, and anti-persistent behavior in the long term. Furthermore, the change in slope, from a persistent to an anti-persistent regime, was interpreted as a sign of intermittent control. However, it is important to observe that the system considered here is an inertial system, and that may account for higher-order trends manifest in the diffusion plot, including a change in the observed slope. By means of numerical simulations in MATLAB, we observed – Fig S5 – that it is possible to reproduce a diffusion plot like that of Collins and De Luca, with two regions with different slopes.


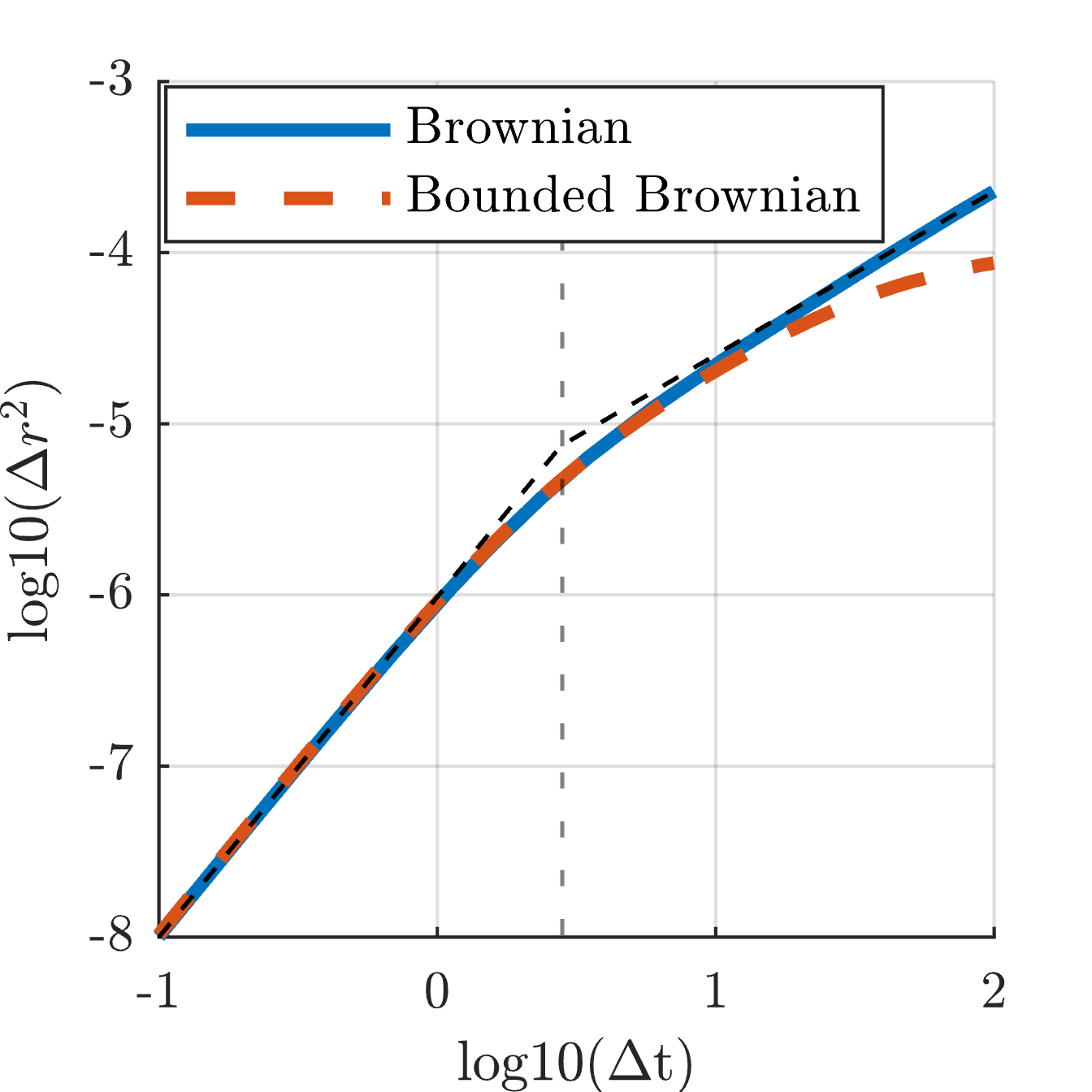


**Fig S5.** **Diffusion plots of a Brownian process (blue line) and of a bounded Brownian process (dashed red line).** Both were filtered through a second-order mechanical system. The vertical dashed grey line indicates the critical point that divides the short-term and long-term regions with different slopes. The black dashed lines represent the best-fit lines to the Brownian process before and after the critical point.

We considered two different inputs: (i) a purely Brownian input and (ii) a bounded Brownian input, obtained by bounding the Brownian process. We then injected these noise sources into a 2^nd^ order dynamical system comprising a mass, spring, and damper. The results – presented in Fig S5 – show the average of 100 simulations performed considering a pseudo-random white noise source with randomized initial seed integrated to obtain a purely Brownian time series that was filtered through a 2^nd^ order mechanical system with the following properties: 75 kg mass, 245 Ns/m damping factor, and 200 N/m stiffness. When we consider a purely Brownian process input (blue line of Fig S3), the slope of the long-term region is exactly 1, corresponding to $H=0.5$. On the other hand, when a bounded Brownian input (dashed red line of Fig S3) was injected in the system, the slope of the long-term region decreased such that $H<0.5$. The slope of short-term region was 2 ($H=1$) with both inputs (purely Brownian and bounded Brownian), and likely arose from inertial dynamics, which is expected to exhibit “persistent” behavior. These results also agree with the spectral analysis of the experimental data: the Brownian process dominates at lower frequencies (long time scales), with flatter slopes observed when the process is bounded.

**Confidence Intervals**

A linear regression model was used to fit the low-frequency region of the PSDs of the analyzed motor tasks. The linear regression was performed in the log-log plane, on the Bode magnitude plot of the PSDs. For each subject a best-fit slope $m_{sl}$ [dB/dec] and a best-fit intercept $q_{sl}$ [dB] was identified such that:

$$PSD=m_{sl}\cdot f+q_{sl}$$

Additionally, for each regression line, the coefficient of determination $R^{2}$ was computed to assess how well a linear model fit the low-frequency data.

The confidence intervals (CI) on the identified slope $m_{sl}$ of the PSD were computed using the following equation:

$$CI= m_{sl}+t\left( \left[ \alpha,1-\alpha\right],N-2 \right)\cdot\left( \frac{\sigma}{\sqrt{N-2}} \right)$$

$$\alpha=\frac{1}{2}\frac{100-CI_{target}}{100}$$

where $t\left( \left[ \alpha,1-\alpha\right],N-2 \right)$ represents the t-score cumulative distributed function values for a confidence range of $\alpha$. $N$ represents the number of samples, while $\sigma$ is the standard deviation of the slope [dB/dec]. We set the confidence interval target at $CI_{target}=95\%$.

The standard deviation of the slope $\sigma$ was computed as:

$$\sigma=\sqrt{\frac{\sum_{i=1}^{N} \left( PSD_{m}\left( i \right)-PSD_{e}\left( i \right) \right)^{2}}{\sum_{i=1}^{N} \left( {\log_{10} f_{m}(i)}_{m}-mean\left( \log_{10} f_{m}(i) \right) \right)^{2}}}$$

where $PSD_{m}$ is the measured power spectral density, $PSD_{e}$ is the estimated power spectral density obtained from the linear regression model, and $f_{m}$ is the measured frequency.

The data presented in the main text showed the performance of only one subject for each analyzed condition. Here we report the 95% confidence intervals (CIs) for the identified slope $m_{sl}$ for the Bode-magnitude PSD of each subject for each motor tasks: crank turning (Fig S6), hand posture (Fig S7), and quiet standing (Fig S8). Moreover, Table S1, Table S2, and Table S3 report respectively the $R^{2}$ coefficients for the PSD linear fitting of crank turning, hand posture, and quiet standing.


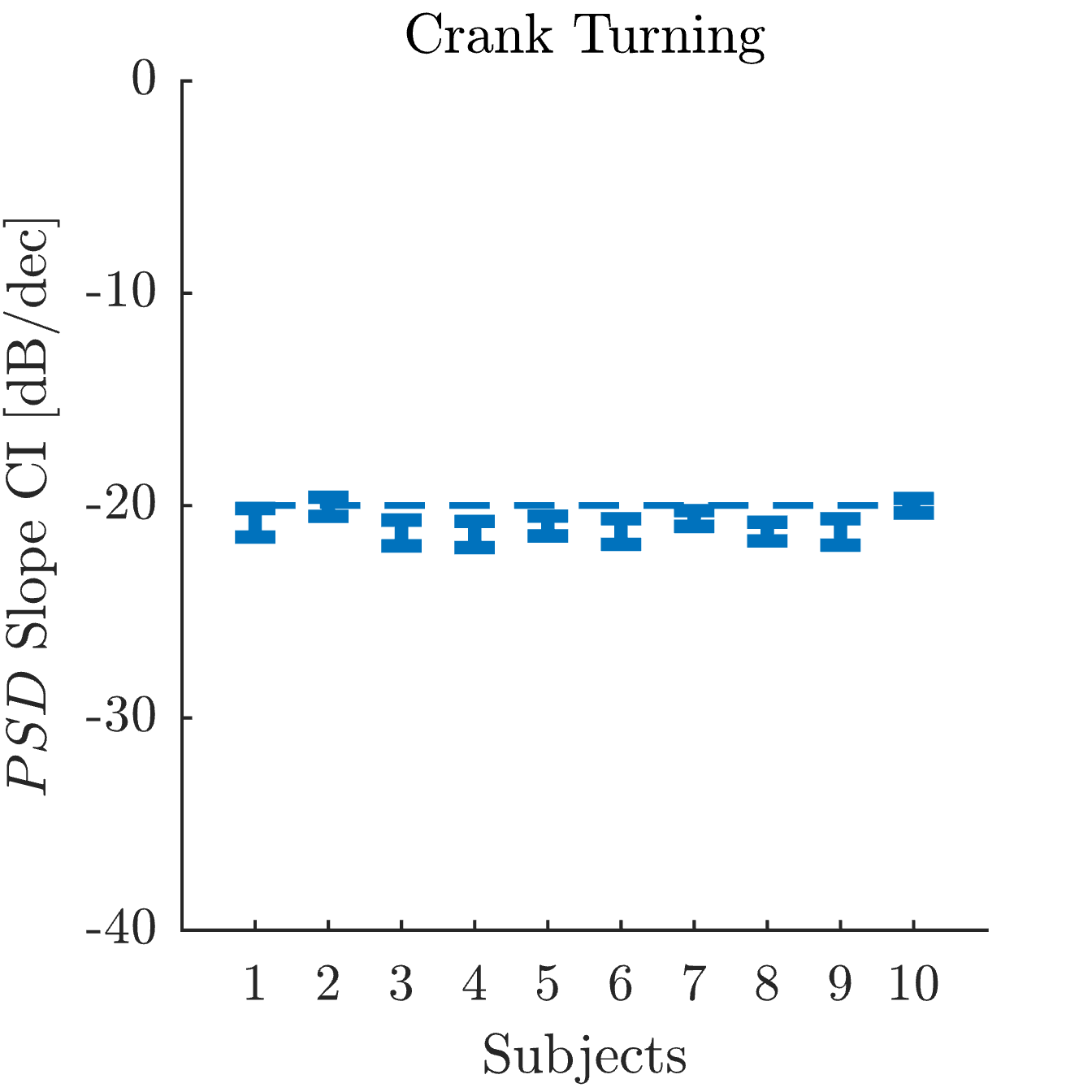


**Fig S6.** **Confidence Intervals (CI) of the best-fit slope for the Bode-magnitude PSD of the crank angular position for each tested subject during the crank-turning experiment.**


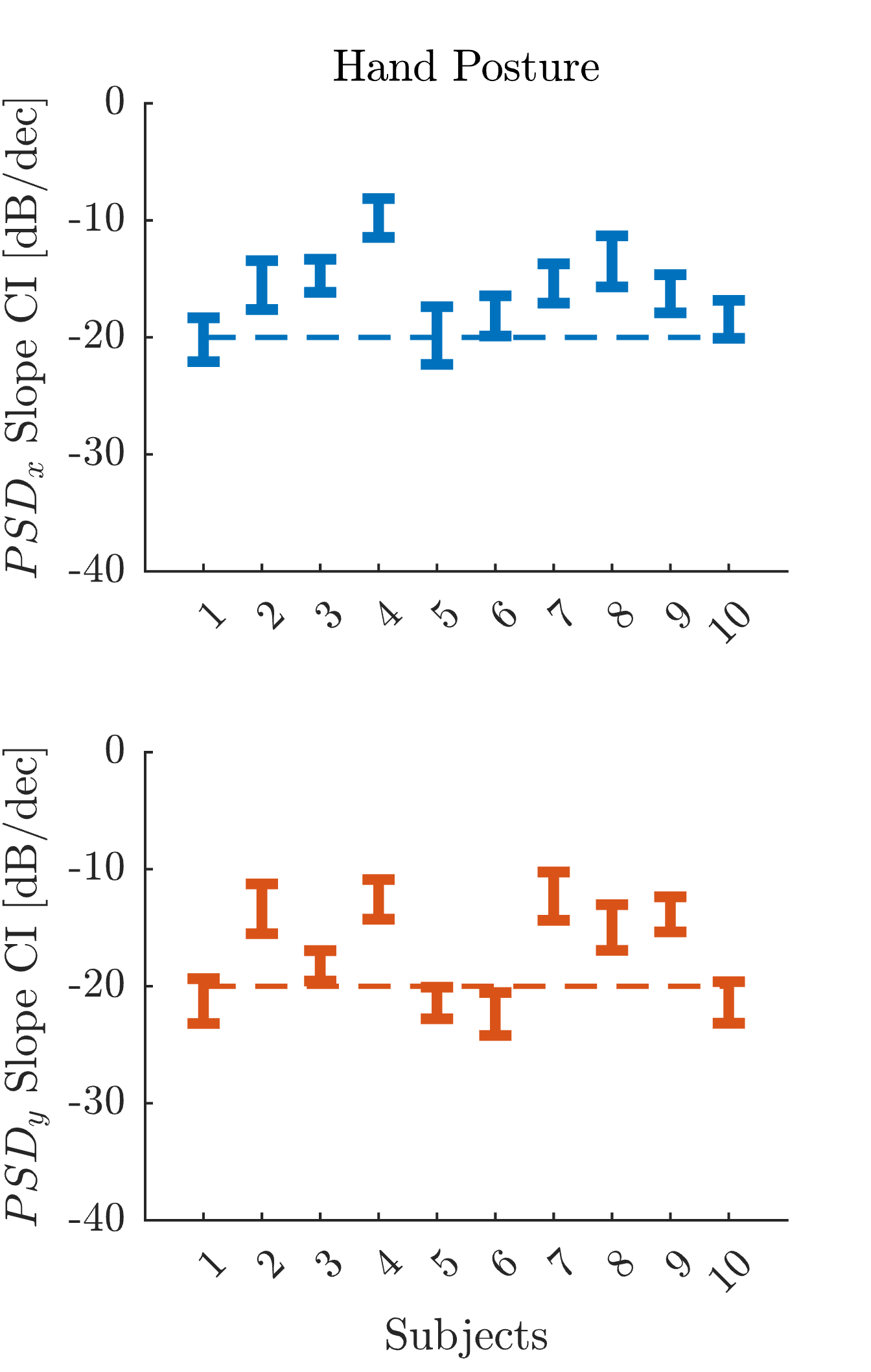


**Fig S7.** **Confidence Intervals (CI) of the best-fit slope for the Bode-magnitude PSD of the hand Cartesian position for each tested subject during the hand-posture experiment.** The top chart shows the ‘x’ component of the hand trajectory, while the bottom chart shows the ‘y’ component of the hand trajectory.


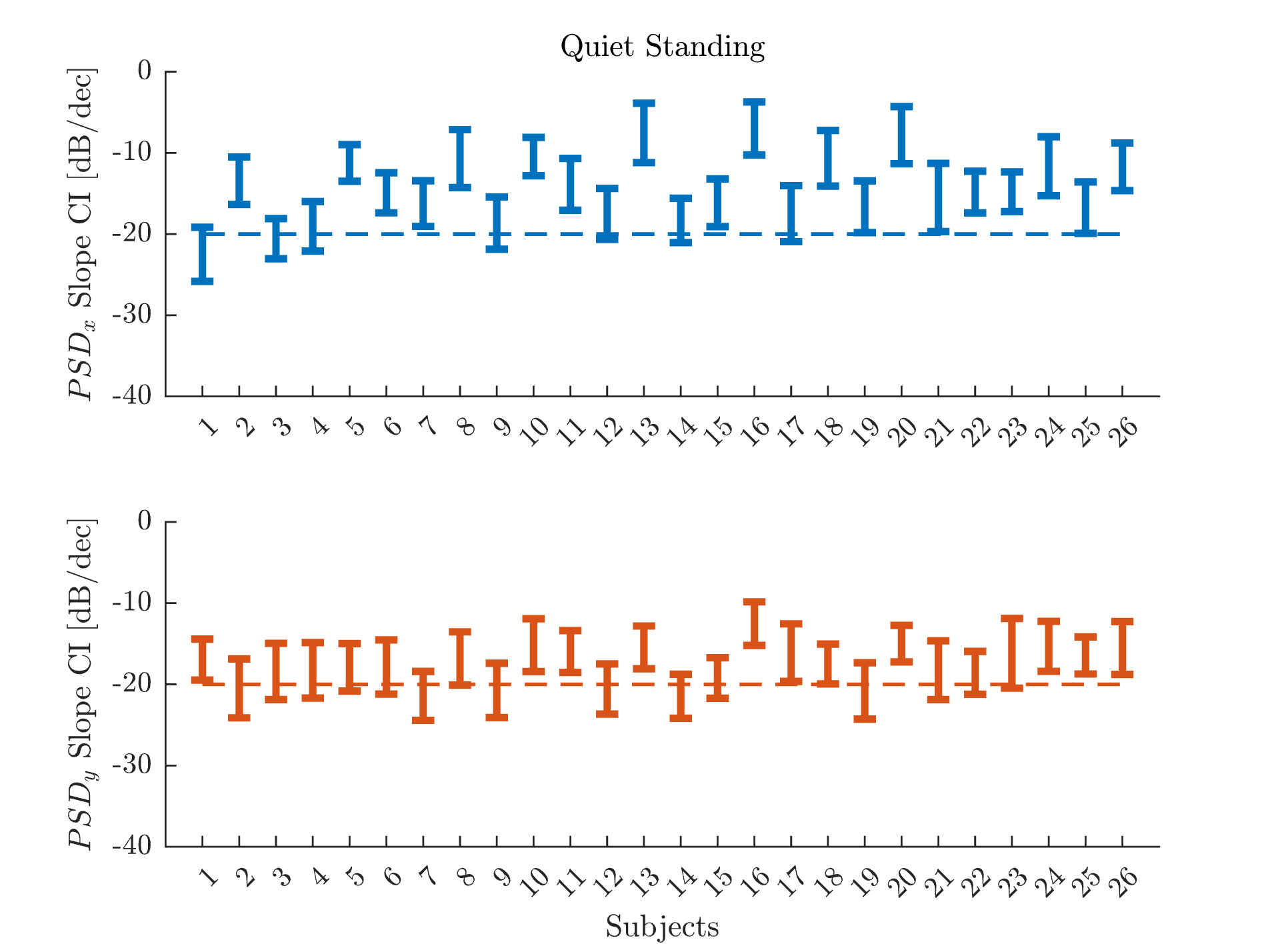


**Fig S8.** **Confidence Intervals (CI) of the best-fit slope for the Bode-magnitude PSD of the center of mass (CoM) trajectory for each tested subject during the quiet standing experiment.** The top chart shows the ‘x’-component of the CoM position, while the bottom chart shows the ‘y’-component of the CoM position.

**Table S1.** **Linear regression** $\boldsymbol{R}^{\boldsymbol{2}}$ **coefficients of the low-frequency PSD fit during crank turning in different subjects.**

| Subject | PSD – Linear Regression $R^{2}$ |  |
| --- | --- | --- |
| $1$ | 0.988 |  |
| $2$ | 0.994 |  |
| $3$ | 0.991 |  |
| 4 | 0.990 |  |
| 5 | 0.994 |  |
| 6 | 0.991 |  |
| 7 | 0.996 |  |
| 8 | 0.995 |  |
| 9 | 0.990 |  |
| 10 | 0.997 |  |
| TOT | 0.993$\pm$0.003 |  |

**Table S2.** **Linear regression** $\boldsymbol{R}^{\boldsymbol{2}}$ **coefficients of the low-frequency PSD fit during hand posture in the different subjects for both ‘x’ and ‘y’ directions.**

| Subject | Direction | PSD – Linear Regression $R^{2}$ |
| --- | --- | --- |
| $1$ | $x$ | 0.92 |
|  | $y$ | 0.92 |
| $2$ | $x$ | 0.80 |
|  | $y$ | 0.74 |
| $3$ | $x$ | 0.92 |
|  | $y$ | 0.95 |
| 4 | $x$ | 0.65 |
|  | $y$ | 0.75 |
| 5 | $x$ | 0.87 |
|  | $y$ | 0.96 |
| 6 | $x$ | 0.92 |
|  | $y$ | 0.94 |
| 7 | $x$ | 0.86 |
|  | $y$ | 0.72 |
| 8 | $x$ | 0.79 |
|  | $y$ | 0.85 |
| 9 | $x$ | 0.91 |
|  | $y$ | 0.89 |
| 10 | $x$ | 0.93 |
|  | $y$ | 0.94 |
| TOT |  | 0.86$\pm0.09$ |

**Table S3.** **Linear regression** $\boldsymbol{R}^{\boldsymbol{2}}$ **coefficients of the low-frequency PSD fit during quiet standing in the different subjects for both ‘x’ and ‘y’ directions.**

| Subject | Direction | PSD – Linear Regression $R^{2}$ | Subject | Direction | PSD – Linear Regression $R^{2}$ |
| --- | --- | --- | --- | --- | --- |
| $1$ | $x$ | 0.81 | $1$4 | $x$ | 0.81 |
|  | $y$ | 0.81 |  | $y$ | 0.86 |
| $2$ | $x$ | 0.67 | $15$ | $x$ | 0.74 |
|  | $y$ | 0.75 |  | $y$ | 0.85 |
| $3$ | $x$ | 0.87 | $16$ | $x$ | 0.30 |
|  | $y$ | 0.73 |  | $y$ | 0.67 |
| 4 | $x$ | 0.79 | 17 | $x$ | 0.71 |
|  | $y$ | 0.73 |  | $y$ | 0.66 |
| 5 | $x$ | 0.70 | 18 | $x$ | 0.48 |
|  | $y$ | 0.78 |  | $y$ | 0.83 |
| 6 | $x$ | 0.78 | 19 | $x$ | 0.72 |
|  | $y$ | 0.73 |  | $y$ | 0.77 |
| 7 | $x$ | 0.76 | 20 | $x$ | 0.32 |
|  | $y$ | 0.83 |  | $y$ | 0.81 |
| 8 | $x$ | 0.46 | 21 | $x$ | 0.56 |
|  | $y$ | 0.71 |  | $y$ | 0.71 |
| 9 | $x$ | 0.76 | 22 | $x$ | 0.76 |
|  | $y$ | 0.78 |  | $y$ | 0.82 |
| 10 | $x$ | 0.65 | 23 | $x$ | 0.78 |
|  | $y$ | 0.67 |  | $y$ | 0.57 |
| 11 | *x* | 0.64 | 24 | *X* | 0.49 |
|  | *y* | 0.78 |  | *Y* | 0.70 |
| 12 | *x* | 0.75 | 25 | *X* | 0.72 |
|  | *y* | 0.81 |  | *Y* | 0.83 |
| 13 | *x* | 0.29 | 26 | *X* | 0.60 |
|  | *y* | 0.77 |  | *Y* | 0.68 |
|  |  |  | TOT |  | 0.70$\pm$0.14 |

**Linear Regressions of Variance**

For each subject in the crank-turning and hand-posture data, the variance trend with respect to time was computed across trials – see the Methods section for details of the computation. For each motor task and for each subject, a linear regression model was fit to the variance. In the crank-turning task, the linear model was fit over the entire trial duration. In the hand-posture task, two best-fit lines were fit: (i) one for the initial linearly growing variance, until the breakpoint was reached ($t_{bp}$), and (ii) a second one for the entire trial duration. The breakpoint time $\left( t_{bp} \right)$ was identified as the time presenting the maximum linear fitting performance. This was found by computing the linear regression for increasing time windows and identifying the time window at which $R^{2}$ reached a maximum value, after which it declined. The search was performed in increments of $\Delta t=1 s$, spanning from a minimum window of $1 s$ to a maximum window of $200 s$. Tables S4 and S5 report the $R^{2}$ coefficients for each subject respectively in the crank-turning and hand-posture tasks. Table S5 also reports the breakpoint time for each subject.

| Subject | Variance – Linear Regression $R^{2}$ |  |
| --- | --- | --- |
| $1$ | 0.96 |  |
| $2$ | 0.96 |  |
| $3$ | 0.96 |  |
| 4 | 0.97 |  |
| 5 | 0.98 |  |
| 6 | 0.96 |  |
| 7 | 0.96 |  |
| 8 | 0.99 |  |
| 9 | 0.91 |  |
| 10 | 0.98 |  |
| TOT | 0.96$\pm$0.02 |  |

**Table S4.** **Linear regression** $\boldsymbol{R}^{\boldsymbol{2}}$ **coefficients for fits to the variance over time during crank turning in different subjects.**

**Table S5.** **Linear regression** $\boldsymbol{R}^{\boldsymbol{2}}$ **coefficients for fits to the variance over time during the hand-posture task in the different subjects for both ‘x’ and ‘y’ directions.** The table reports the $R^{2}$ for both the initial part with linearly growing variance until the breakpoint $(t_{bp})$ and for the variance over the entire trial duration.

| Subject | Direction | Variance – Linear Regression $R^{2}$ $(t\leq t_{bp})$ | $t_{bp} [s]$ | Variance – Linear Regression $R^{2}$ $(\forall t)$ |
| --- | --- | --- | --- | --- |
| $1$ | $x$ | 0.92 | 75.00 | 0.38 |
|  | $y$ | 0.95 | 50.00 | 0.07 |
| $2$ | $x$ | 0.83 | 4.00 | 0.00 |
|  | $y$ | 0.83 | 5.00 | 0.00 |
| $3$ | $x$ | 0.64 | 16.00 | 0.02 |
|  | $y$ | 0.91 | 7.00 | 0.58 |
| 4 | $x$ | 0.95 | 0.99 | 0.03 |
|  | $y$ | 0.97 | 2.00 | 0.01 |
| 5 | $x$ | 0.81 | 136.00 | 0.30 |
|  | $y$ | 0.95 | 38.00 | 0.35 |
| 6 | $x$ | 0.87 | 42.00 | 0.22 |
|  | $y$ | 0.76 | 35.00 | 0.04 |
| 7 | $x$ | 0.90 | 0.99 | 0.05 |
|  | $y$ | 0.88 | 10.00 | 0.08 |
| 8 | $x$ | 0.76 | 19.00 | 0.00 |
|  | $y$ | 0.75 | 44.00 | 0.00 |
| 9 | $x$ | 0.69 | 10.00 | 0.05 |
|  | $y$ | 0.80 | 16.00 | 0.01 |
| 10 | $x$ | 0.93 | 108.00 | 0.00 |
|  | $y$ | 0.95 | 61.00 | 0.06 |
| TOT |  | 0.85$\pm0.10$ |  | $0.11\pm0.16$ |

1. The variance was normalized in order to provide a better visual comparison between the different simulated time constants $\tau_{d}$. [↑](#footnote-ref-1)
